# Studies on enmetazobactam clarify mechanisms of widely used β-lactamase inhibitors

**DOI:** 10.1073/pnas.2117310119

**Published:** 2022-04-29

**Authors:** Pauline A. Lang, Ritu Raj, Anthony Tumber, Christopher T. Lohans, Patrick Rabe, Carol V. Robinson, Jürgen Brem, Christopher J. Schofield

**Affiliations:** ^a^Chemistry Research Laboratory, Department of Chemistry, University of Oxford, Oxford OX1 3TA, United Kingdom;; ^b^Ineos Oxford Institute for Antimicrobial Research, University of Oxford, Oxford OX1 3TA, United Kingdom;; ^c^Physical and Theoretical Chemistry Laboratory, Department of Chemistry, University of Oxford, Oxford OX1 3TA, United Kingdom;; ^d^Kavli Institute for Nanoscience Discovery, University of Oxford, Oxford OX1 3QU, United Kingdom

**Keywords:** enmetazobactam, tazobactam, serine β-lactamase inhibitor, antimicrobial resistance, mechanism-based inhibition

## Abstract

β-Lactams are the most important class of antibacterials, but their use is increasingly compromised by resistance, most importantly via serine β-lactamase (SBL)-catalyzed hydrolysis. The scope of β-lactam antibacterial activity can be substantially extended by coadministration with a penicillin-derived SBL inhibitor (SBLi), i.e., the penam sulfones tazobactam and sulbactam, which are mechanism-based inhibitors working by acylation of the nucleophilic serine. The new SBLi enmetazobactam, an *N*-methylated tazobactam derivative, has recently completed clinical trials. Biophysical studies on the mechanism of SBL inhibition by enmetazobactam reveal that it inhibits representatives of all SBL classes without undergoing substantial scaffold fragmentation, a finding that contrasts with previous reports on SBL inhibition by tazobactam and sulbactam. We therefore reinvestigated the mechanisms of tazobactam and sulbactam using mass spectrometry under denaturing and nondenaturing conditions, X-ray crystallography, and NMR spectroscopy. The results imply that the reported extensive fragmentation of penam sulfone–derived acyl–enzyme complexes does not substantially contribute to SBL inhibition. In addition to observation of previously identified inhibitor-induced SBL modifications, the results reveal that prolonged reaction of penam sulfones with SBLs can induce dehydration of the nucleophilic serine to give a dehydroalanine residue that undergoes reaction to give a previously unobserved lysinoalanine cross-link. The results clarify the mechanisms of action of widely clinically used SBLi, reveal limitations on the interpretation of mass spectrometry studies concerning mechanisms of SBLi, and will inform the development of new SBLi working by reaction to form hydrolytically stable acyl–enzyme complexes.

## Significance

Microbial resistance to β-lactam antibiotics mediated by β-lactamase–catalyzed hydrolysis is a major global health concern. Penam sulfones, which are structurally related to penicillins, inhibit clinically important serine β-lactamases (SBLs) by forming transiently stable covalent complexes, thereby protecting β-lactam antibiotics from hydrolysis. The characterization of these complexes and mechanisms of SBL inhibition is important for development of new SBL inhibitors (SBLi). Studies on the mechanism of the new SBLi enmetazobactam employing mass spectrometry and X-ray crystallography inform on its mode of action and also lead to reevaluation of mechanisms of current clinically important SBLi. In addition to insights into the mechanisms of transient SBL inhibition by penam sulfones, the results reveal potential for penam sulfone optimization to enable irreversible SBL inhibition.

**Fig. 1.**
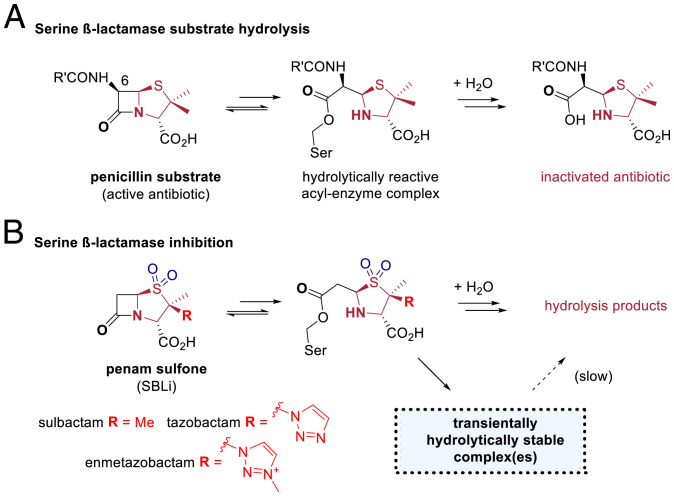
Sulfone derivatives of penicillins are potent clinically used mechanism-based inhibitors of SBLs. (*A*) Outline mechanism for penicillin hydrolysis as catalyzed by SBLs; reaction proceeds via an AEC, which is efficiently hydrolyzed. (*B*) Sulfone derivatives of penicillins are SBLi that react to give one or more hydrolytically stable complex(es), the nature of which was the focus of our work.

**Fig. 2.**
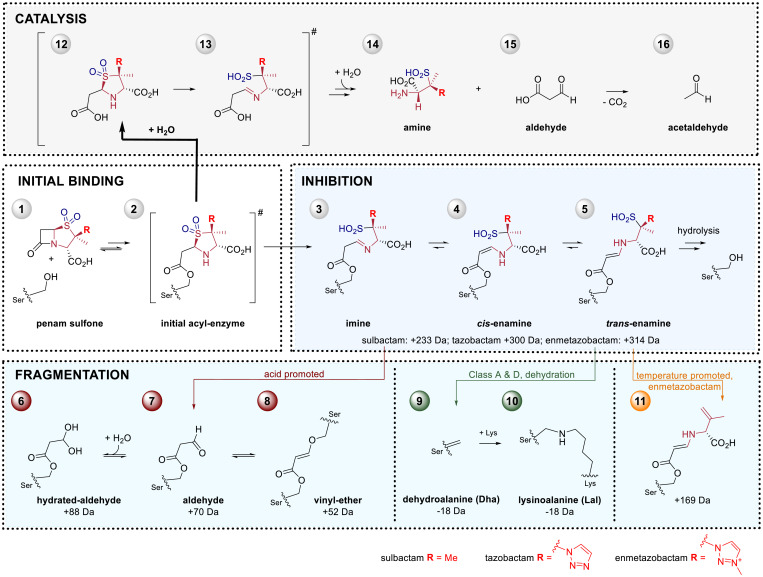
Pathways for reactions of penam sulfones with SBLs. Following initial acyl–enzyme **2** formation the main transient inactivation pathway occurs via thiazolidine ring opening to give species **3**-**5** which are relatively stable to hydrolysis. Fragmentation of **3**-**5** can occur in rare cases and is promoted by acid to give **6**-**8** or heat to give **11**. In rare cases fragmentation of **2**-**5** can result in irreversible inactivation of the SBL to give **9** and **10**. Efficient hydrolysis of the β-lactam occurs to give a β-amino acid product **12**, which in solution fragments to give **13**-**16**. Our results imply biologically relevant inhibition involves **3**-**5**, or equivalent mass species.

β-Lactamases are a major mechanism of resistance to the clinically vital β-lactam antibiotics, with >2,000 different β-lactamases reported (1). β-Lactamases are grouped into classes A, C, and D, which employ a nucleophilic serine in catalysis (serine β-lactamases, SBLs), and class B, which employ metal ions in catalysis (2). Presently, SBLs are the most important β-lactamases from a clinical perspective. SBL inhibitors (SBLi) have been developed for use in combination with a β-lactam antibiotic, with tazobactam (3), sulbactam (4), and clavulanic acid (5) being the most widely used SBLi. These SBLi all contain a β-lactam ring which reacts with SBLs to produce an acyl–enzyme complex (AEC) intermediate, as is also the case for efficient SBL substrates (Fig. 1*A*). With efficient substrates the β-lactam–derived AEC is readily hydrolyzed. With SBLi the reaction bifurcates at the AEC stage; in addition to hydrolysis, reaction of the AEC via opening of the β-lactam fused five-membered ring occurs to give one or more relatively hydrolytically stable species (Figs. 1*B* and 2). The nature of these species is central to SBLi inhibition and has been studied by crystallography (6–11) and ultraviolet-visible (UV/Vis) (10, 12) and Raman (6, 7, 9, 12–15) spectroscopy, as well as different types of mass spectrometry (MS) (10, 16–22).

The structures of tazobactam and sulbactam are closely related to those of the penicillins; they differ by lack of a C-6 side chain, functionalization of the *pro-S* methyl group (in case of tazobactam), and by oxidation of the thiazolidine to a sulfone. These differences result in a loss of useful antibacterial activity but a gain of potent SBL inhibition. Although the presence of sulfur in drugs is common [e.g., sulfonamide antibiotics (23)] and there is growing interest in covalently acting drugs (24, 25), sulfones are rare in drugs and, as far as we are aware, sulbactam and tazobactam are the only clinically approved sulfone-containing drugs working by covalent reaction with their targets (26–28).

Since the clinical introduction of the pioneering SBLi, β-lactamases have evolved and SBLi use is increasingly compromised by extended spectrum β-lactamases (ESBLs) and inhibitor-resistant SBLs (29). Efforts have been made to develop new SBLi, including those with and without a β-lactam. The latter include diazabicyclooctanes (30) and cyclic boronates (31, 32). However, β-lactam–containing SBLi remain of most clinical importance. Among SBLi in clinical development, enmetazobactam (formerly AAI-101; Fig. 1) is of particular interest because it is a “simple” *N*-methylated derivative of the triazole ring of tazobactam (33). In combination with cefepime, enmetazobactam is reported to manifest substantially better antimicrobial properties against class A ESBL-producing strains than the commonly used piperacillin/tazobactam combination (20, 33, 34).

We report studies on the mechanism of SBL inhibition by enmetazobactam using denaturing and nondenaturing (native) MS methods, NMR spectroscopy, and crystallography. The results led us to reevaluate the mechanisms of SBL inhibition by the clinically important sulfone-containing SBLi, i.e., tazobactam and sulbactam, and reveal limitations on the interpretation of MS studies concerning SBL inhibition.

## Results

Initially, we studied inhibition of representative clinically important SBLs, i.e., TEM-116 (class A), AmpC from *Escherichia coli* (class C, AmpC*_EC_*), and OXA-10 (Class D), by enmetazobactam and, for comparison, tazobactam and sulbactam, by fluorescence spectroscopy using the reporter substrate FC-5 (35) (*SI Appendix*, Figs. S1–S3). After 10 min of preincubation with the inhibitors, TEM-116 was potently inhibited by enmetazobactam (concentration that inhibits response by 50% [IC_50_]: 36 nM), tazobactam (IC_50_: 11 nM), and sulbactam (IC_50_: 590 nM) (*SI Appendix*, Table S1 and Fig. S1). AmpC*_EC_* showed much weaker inhibition (IC_50_s, enmetazobactam: 81 μM; tazobactam 92 μM; sulbactam: 89 μM) (*SI Appendix*, Table S2 and Fig. S2). Inhibition of OXA-10 [± NaHCO_3_ to ensure Lys70 carbamylation (36)] by enmetazobactam (IC_50_: 5.9 μM) and tazobactam (IC_50_: 1.9 μM) was moderate, with weaker inhibition by sulbactam (IC_50_: 143 μM) (*SI Appendix*, Table S1 and Fig. S1). For AmpC*_EC_* and OXA-10, the potency of inhibition depended on the preincubation time, while for TEM-116 varying the preincubation time had no apparent impact (*SI Appendix*, Fig. S3).

More detailed kinetic analyses (*SI Appendix*, Figs. S4–S7 and Table S4) revealed significantly slower inactivation rates (*k*_inact_/*K* or *k*_2_/K) of the penam sulfones with OXA-10 (138 ± 7 M⋅s^−1^, 42 ± 2 M⋅s^−1^, and 3.8 ± 0.1 M⋅s^−1^ for tazobactam, enmetazobactam, and sulbactam, respectively) compared to TEM-116 (746 ± 32 × 10^3^ M⋅s^−1^, 209 ± 5 × 10^3^ M⋅s^−1^, and 13.1 ± 0.1 × 10^3^ M⋅s^−1^, respectively); the dissociation rates (*k*_off_) and half-lives of the inhibited species (*t*_1/2_) were similar (2 to 11 min). The estimated partition ratios between acyl–enzyme hydrolysis and transient inhibition were higher for OXA-10 (160, 1,700, and 5,300 for tazobactam, enmetazobactam, and sulbactam, respectively) compared to TEM-116 (9, 4, and 240, respectively). With AmpC*_EC_* very slow dissociation rates were observed (with *t*_1/2_ 1,746 ± 10 min, 803 ± 2 min, and 616 ± 1 min for tazobactam, enmetazobactam, and sulbactam, respectively). However, the inactivation rates for AmpC*_EC_* were lower (7.6 ± 0.6 M⋅s^−1^, 4.1 ± 0.5 M⋅s^−1^, and 4.9 ± 0.3 M⋅s^−1^, respectively) and the partition ratios (988, 5,100, and 3,100, respectively) substantially higher than with OXA-10 and TEM-116, rationalizing the lower potency of the penam sulfones against AmpC*_EC_*.

To investigate the products of the reactions of enmetazobactam and SBLs we employed protein observed positive ion electrospray ionization (ESI) MS. We developed a solid-phase extraction linked to MS (SPE-MS) based method, in anticipation it would be useful for high-throughput assays. Compared to conventional liquid chromatography-MS (LC-MS) techniques, SPE-MS uses cartridges that manifest nonoptimal separation but which require shorter elution times (4 s compared to 3 to 10 min). The sample is applied to a cartridge, washed with 5% (vol/vol) aqueous acetonitrile (4 s), then eluted directly into the spectrometer in 95% (vol/vol) aqueous acetonitrile. As is standard procedure, to protonate proteins for positive ion ESI-MS analysis in both LC-MS and SPE-MS methods an organic acid was added to the solvents (0.1% (vol/vol) formic acid).

**Fig. 3.**
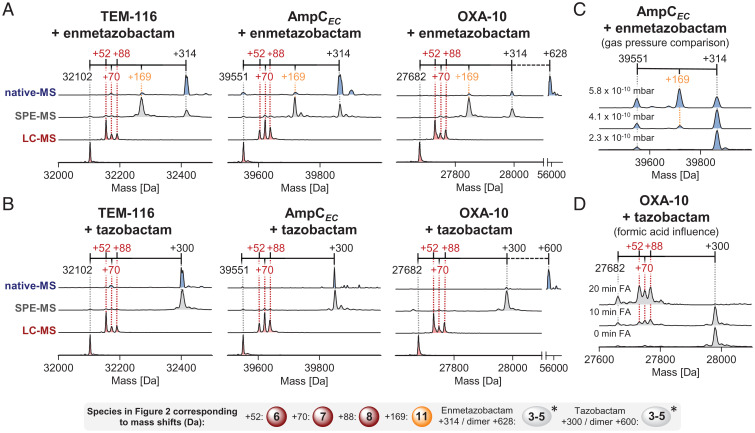
The method of mass spectrometric analysis influences the products observed on reaction of penam sulfones with SBLs. Results of protein-observed LC-, SPE-, and nondenaturing- (native) MS analysis of reactions of SBLs with (*A*) enmetazobactam and (*B*) tazobactam are shown. For LC-MS, TEM-116, AmpC*_EC_*, or OXA-10 (1 μM) were incubated with tazobactam or enmetazobactam (100 μM, 50 mM Tris, pH 7.5, 20 min, r.t.). For SPE-MS, TEM-116, AmpC*_EC_*, or OXA-10 (3 μM) were incubated with tazobactam or enmetazobactam (300 μM, 50 mM Tris, pH 7.5, 20 min, r.t.). For LC-MS, protein elution used a gradient of 5 to 95% acetonitrile with 0.1% (vol/vol) aqueous formic acid (over ∼4 to 5 min); elution with SPE-MS employed the same mobile phase (within 4 s). For native MS, AmpC*_EC_*, OXA-10, or TEM-116 (10 μM) were incubated with tazobactam or enmetazobactam (200 μM, 1M ammonium acetate, pH 7.5, 20 min, r.t.), desalted, then sprayed into the spectrometer. As reported (70), OXA-10 was predominantly dimeric. (*C*) Native-MS results with enmetazobactam derived AmpC*_EC_* complexes vary with spectrometer gas pressure. *SI Appendix*, Figs. S9–S13 show nondeconvoluted native-MS spectra. (*D*) SPE-MS studies with tazobactam-derived OXA-10 complexes showing the effect of adding 0.1% (vol/vol) formic acid (FA). *SI Appendix*, Table S5 summarizes calculated and observed masses. *Note mass shifts may reflect more than one structure, e.g. **2**-**5** (Fig. 2) give the same mass shift.

By contrast with reported results for enmetazobactam using a conventional LC-MS protocol (20), the SPE-MS analysis of covalent modifications of TEM-116, AmpC*_EC_*, and OXA-10 after incubation with enmetazobactam manifested a +314-Da mass increment after 20 min, consistent with formation of species with the full inhibitor mass (e.g., enamine **5** or equivalent mass species; Figs. 2 and 3). A +169-Da mass increment was also observed, likely corresponding to the product of elimination from an imine or enamine intermediate (see below). Only very weak peaks for masses corresponding to the reported (20) further fragmented products, with +52-, +70-, and +88-Da mass shifts (potentially reflecting formation of **6**, **7**, and **8**; Fig. 2), were detected.

To investigate the apparent discrepancies between our SPE-MS results and the prior study using LC-MS protocols we analyzed reactions of TEM-116, AmpC*_EC_*, and OXA-10 with 100 equivalents of enmetazobactam using an LC-MS method similar to that reported (20). A clear difference between the SPE-MS and LC-MS results for enmetazobactam was manifest, with LC-MS analyses showing complete conversion to the fragmented +52-, +70-, and +88-Da species (likely reflecting formation of **6**, **7**, and **8**; Fig. 2) after SBL incubations with a 100-fold excess of enmetazobactam for 20 min (Fig. 3*A*). These observations are consistent with earlier LC-MS studies on sulbactam and tazobactam (10, 16–22).

One difference between the SPE- and LC-MS techniques is the time that the samples are exposed to formic acid. We observed that addition of 0.1% (vol/vol) aqueous formic acid to enmetazobactam-reacted samples under partially buffered conditions (50 mM Tris, pH 7.5) prior to SPE-MS analysis promoted AEC fragmentation with OXA-10 and TEM-116 to give +52-, +70-, and +88-Da species (Fig. 3*D* and *SI Appendix*, Fig. S8); for AmpC*_EC_* it led to accelerated hydrolysis of the AEC (*SI Appendix*, Fig. S8). In the acid-treated samples, the −18-Da modifications produced by reaction of enmetazobactam with OXA-10 and TEM-116 were either not observed or were observed to a lesser extent (*SI Appendix*, Fig. S8).

To further investigate fragmentation of the enmetazobactam-derived +314-Da species to give a +169-Da species, we carried out native ESI-MS experiments, which do not require acid-promoted protonation of proteins and allow for analyses using a lower energy input. Samples produced by incubation at room temperature for 10 to 30 min predominantly manifested a +314-Da species (potentially reflecting formation of *trans*-enamine **5** or equivalent mass species), with only low amounts of the +169-Da species being observed for all SBLs (Fig. 3*B* and *SI Appendix*, Figs. S9–S12). Further analyses using AmpC*_EC_* as a model system revealed correlation between levels of the +169-Da species with the gas pressure (Fig. 3*C* and *SI Appendix*, Fig. S12) and collision energy (*SI Appendix*, Fig. S13) in the spectrometer. The +169-Da species (at low levels) was also observed on incubation of the AmpC*_EC_*-derived AEC at elevated temperature (*SI Appendix*, Fig. S14).

**Fig. 4.**
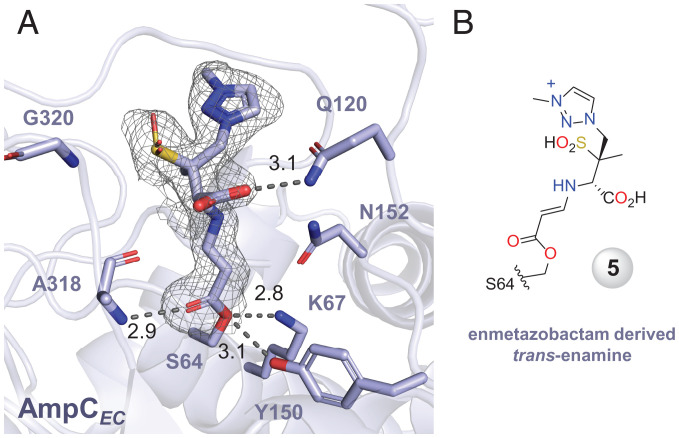
Structural basis of AmpC*_EC_* inhibition by enmetazobactam. (*A*) View from a crystal structure of AmpC*_EC_* reacted with enmetazobactam for 14 min (PDB: 6T35, 1.75-Å resolution), showing the *trans*-enamine species **5** covalently bonded to the nucleophilic Ser64. mF_o_-DF_c_ polder OMIT maps (40) contoured at 3.0 σ and carved around the enmetazobactam derived *trans*-enamine. (*B*) The enmetazobactam derived *trans*-enamine.

Consistent with the native-MS studies, a crystal structure of AmpC*_EC_* acylated by enmetazobactam (1.75-Å resolution, Protein Data Bank [PDB]: 6T35; *SI Appendix*, Table S6), obtained by soaking (400 equiv. enmetazobactam, 14 min), showed continuous density extending from the nucleophilic Ser64 (Fig. 4 and *SI Appendix*, Fig. S15), which was refined as the *trans*-enamine **5**, as also observed in a reported crystal structure of AmpC*_EC_* reacted with tazobactam [PDB: 6XSF (37)].

**Fig. 5.**
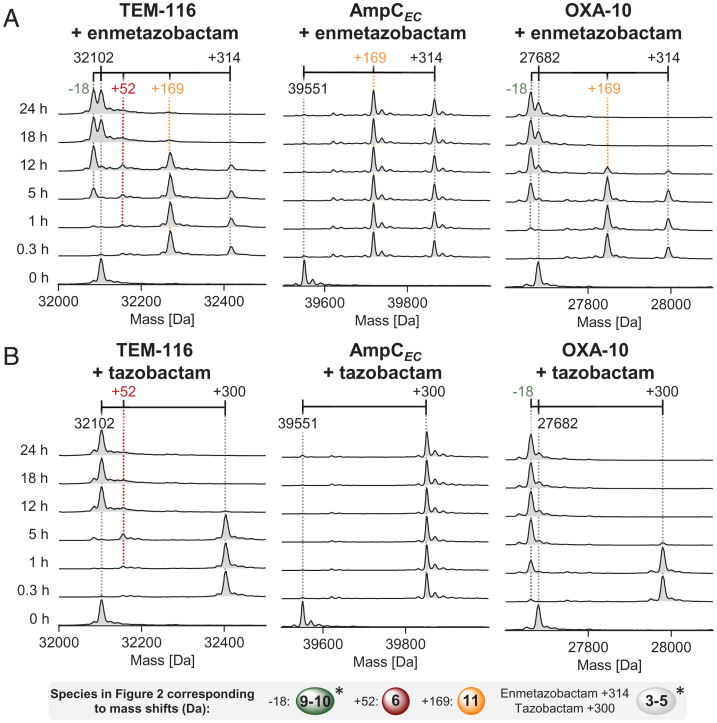
Prolonged reaction of SBLs with tazobactam or enmetazobactam can lead to fragmentation and dehydroalanine formation. SPE-MS analysis of TEM-116, AmpC*_EC_*, or OXA-10 (3 μM) reaction with (*A*) enmetazobactam and (*B*) tazobactam (300 μM inhibitor, in 50 mM Tris, pH 7.5, r.t.). *SI Appendix*, Table S5 summarizes calculated/observed masses/mass shifts. *Note mass shifts may reflect more than one structure, e.g. **2**-**5** and **9**-**10** (Fig. 2) give the same mass shift.

SPE-MS studies on prolonged reaction over 24 h showed that the +314-Da AmpC*_EC_* modifications were relatively stable (Fig. 5*A* and *SI Appendix*, Fig. S16). With a 10-fold excess of enmetazobactam, the unmodified forms of OXA-10 and TEM-116 were fully recovered after 3 h. With TEM-116 and a 100-fold excess of enmetazobactam, a +52-Da mass increment species was observed to accumulate over 12 h, but this was not evident after 18 h. After 18 h (with likely near-complete hydrolysis of the 100 equivalents of enmetazobactam), TEM-116 and OXA-10 both showed an additional species corresponding to a mass decrease of −18-Da relative to the unmodified enzymes, with ∼50% conversion to this species for TEM-116 and ∼70% conversion for OXA-10 (Fig. 5*A* and *SI Appendix*, Fig. S16), indicating conversion in ∼1 out of 200 and ∼1 out of 140 turnover events for TEM-116 and OXA-10, respectively. This observation is consistent with previously proposed loss of water from the nucleophilic serine of the class A SBLs CTX-M-15 and SHV-1 to give a dehydroalanine (Dha) residue **9** (20) or equivalent mass species (see below).

In light of the substantial differences in covalently bound species observed with the different MS methods for SBLs on reaction with enmetazobactam, we reinvestigated the mechanisms of tazobactam and sulbactam. Consistent with prior studies (10, 16–22), with the LC-MS method we observed fragmentation patterns similar to those for enmetazobactam, i.e., full fragmentation to +52-, +70-, and +88-Da species (likely corresponding to **6**, **7**, and **8**; Fig. 2) on incubation with tazobactam (100 equiv.) or sulbactam (500 equiv.) for 20 min (Fig. 3*B* and *SI Appendix*, Fig. S17).

By contrast, and as observed with enmetazobactam, SPE-MS analyses with TEM-116, AmpC*_EC_*, and OXA-10 manifested predominant species with the intact inhibitor mass, i.e., +300-Da shift for tazobactam and +233-Da shift for sulbactam (consistent with formation of e.g., *trans*-enamine **5** or equivalent mass species; Fig. 3*B* and *SI Appendix*, Fig. S17).

Addition of formic acid [0.1% (vol/vol)] to the otherwise stable TEM-116 or OXA-10 complexes derived by reaction with tazobactam or sulbactam (+300- and +233-Da species), under partially buffered conditions (50 mM Tris, pH 7.50), promoted reaction to give the +52-, +70-, and +88-Da mass increment species in less than 1 h, as observed by SPE-MS (Fig. 3*D* and *SI Appendix*, Fig. S18). As observed with enmetazobactam, generation of the −18-Da species was less favored on acid treatment. In the case of AmpC*_EC_*, formic acid addition accelerated regeneration of the unmodified protein (*SI Appendix*, Fig. S18).

In SPE-MS studies on the prolonged reaction of SBLs with the penam sulfones (Fig. 5*B* and *SI Appendix*, Figs. S19 and S20), modifications were observed over a longer time span when using more inhibitor (100 equiv.), with regeneration of the unmodified enzyme being observed with lower amounts of inhibitor (10 equiv.), consistent with bifurcating reactivity of AEC **2** leading to hydrolysis or transient inactivation (Fig. 1*B*). On prolonged incubation (8 h) of TEM-116 and OXA-10 with 100 equiv. of tazobactam, complete conversion to the −18-Da mass shift species was observed by SPE-MS with OXA-10 (suggesting conversion in more than 1 in 100 turnover events), but the −18-Da species was not observed with TEM-116 (Fig. 5*B*). Small amounts of the −18-Da modification were also observed on incubating OXA-10 with 500 equiv. of sulbactam (*SI Appendix*, Fig. S20). Over 5 h low levels of a +52-Da species were observed to accumulate for TEM-116, followed by regeneration of the unmodified enzyme (Fig. 5*B* and *SI Appendix*, Fig. S19).

We investigated products obtained by SBL-catalyzed hydrolysis of enmetazobactam, tazobactam, and sulbactam in solution by ^1^H NMR spectroscopy (750 MHz). In each case, incubation of the inhibitor (100 equiv.) with OXA-10 or TEM-116 manifested efficient turnover in <18 h; AmpC*_EC_* turnover was relatively slow (*SI Appendix*, Figs. S21–S25), consistent with the slower acylation observed in competition studies with FC-5 (*SI Appendix*, Figs. S5–S7) and SPE-MS (*SI Appendix*, Figs. S16, S19, and S20). In all cases the penam sulfone reacted efficiently to give fragmentated products, i.e., amine **14** (which appeared stable over the timescale of analysis) and aldehyde **15** (which underwent further decarboxylation and hydration; *SI Appendix*, Figs. S21–S25).

To further investigate the −18-Da species, native-MS analyses of TEM-116 and OXA-10 samples treated with 100 equiv. of enmetazobactam or tazobactam for 24 h were performed to rule out acid-mediated interference in the generation of the −18-Da modifications; under these conditions the −18-Da modifications were also observed, consistent with the SPE-MS results (*SI Appendix*, Fig. S26). In accord with a previous report (20), −18-Da-modified SBLs showed no β-lactamase activity when assayed with FC-5 (*SI Appendix*, Fig. S27).

**Fig. 6.**
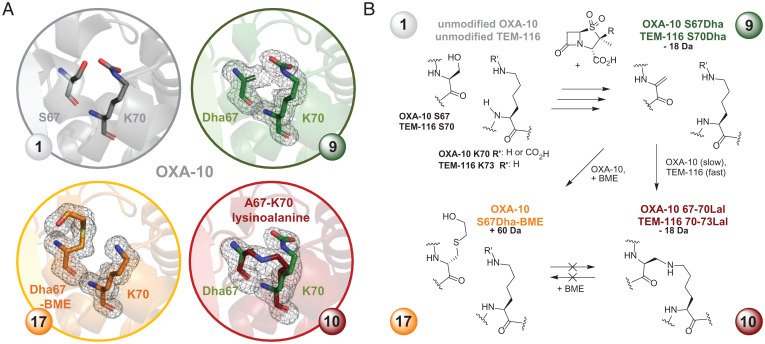
Reaction of SBLs with tazobactam or enmetazobactam produces a dehydroalanine (Dha) residue that reacts to give a cross-linked lysinoalanine (Lal) species. (*A*) Views from a crystal structure of unmodified OXA-10 **1** [PDB: 2X02 (39), 1.35-Å resolution], −18-Da modified OXA-10, from incubation with tazobactam (100 equiv., 18 h, r.t.), showing either S67Dha **9** (PDB: 7B3S, 1.85-Å resolution) or S67Dha OXA-10 **9** together with partial formation of 67-70Lal **10** (PDB: 7B3R, 1.83-Å resolution) and OXA10 S67Dha-BME species **17** (PDB: 7B3U, 1.60-Å resolution), derived from simultaneous incubation of OXA-10 with 100 equiv. tazobactam and 1,000 equiv. BME. mF_o_-DF_c_ polder OMIT maps (40) contoured at 3.0 σ, carved around active site residues from one (chain A) of two chains in the ASU are shown. See *SI Appendix*, Figs. S32 and S33 for mF_o_-DF_c_ polder OMIT maps for chain B. (*B*) Modifications observed on treatment of OXA-10 and TEM-116 with tazobactam, or enmetazobactam, with/without added BME.

To test for formation of Dha-containing species **9**, the −18-Da protein product was subjected to reaction with a thiol, a reaction used in protein engineering to introduce covalent modifications (38). No reaction was observed on incubation of unmodified OXA-10 with β-mercaptoethanol (BME, 1,000 equiv.), as monitored by SPE-MS (*SI Appendix*, Fig. S28). By contrast, after complete modification of OXA-10 to give the −18-Da species (8 h with excess tazobactam; validated by SPE-MS) and subsequent addition of BME (1,000 equiv.), ∼80% of the −18-Da protein was converted to a +60-Da species (relative to unmodified OXA-10) within 2 h. This observation is in agreement with the addition of BME to Dha **9** to give 2-hydroxyethyl-cysteine (Dha-BME, **17**; Fig. 6). Interestingly, this reaction did not progress further, even after overnight incubation with more BME (*SI Appendix*, Fig. S28). The extent of conversion to the +60-Da species **17** was increased to ≥98% by simultaneous incubation of OXA-10 with tazobactam (100 equiv.) and BME (1,000 equiv.) for 12 h and was decreased by prolonged incubation with tazobactam prior to BME addition (*SI Appendix*, Fig. S28). These results imply a slow inactivation of the initially formed Dha residue **9** toward reaction with BME, a proposal rationalized by subsequent studies (see below).

Reaction of unmodified TEM-116 with BME manifested slow addition of a single BME molecule over 24 h (likely via thiol disulfide exchange with either one of the two disulfide-bound Cys residues; *SI Appendix*, Fig. S29). When TEM-116 was first treated with enmetazobactam and then BME, analogous slow addition of a single BME was observed; no addition of a second BME was observed, even on prolonged incubation, as would be expected for reaction of BME with a Dha residue **9** (*SI Appendix*, Fig. S29). By contrast with OXA-10, reactivity of the TEM-116 derived −18-Da modified protein with BME could not be increased by simultaneous incubation of TEM-116 with enmetazobactam and BME. These results suggest that if a Dha residue **9** is formed on TEM-116, inactivation of its reaction with BME proceeds more rapidly than with OXA-10.

MS fragmentation (MS/MS) analysis of trypsin-hydrolyzed SBLi-treated samples of OXA-10 and TEM-116 supported the presence of modification of the nucleophilic Ser. With OXA-10 treated simultaneously with tazobactam and BME, a +60-Da modification (in agreement with Dha-BME **17**) was observed at Ser67 (*SI Appendix*, Fig. S30). Notably, whereas MS/MS analysis of the tryptic peptide containing Ser70 residue from unmodified TEM-116 sample showed complete backbone fragmentation (*SI Appendix*, Fig. S31*A*), no MS/MS fragmentation of the corresponding enmetazobactam-reacted −18-Da modified peptide was observed beyond residues Lys73 or Ser70, consistent with cross-linking of the side chains of these residues (see below; *SI Appendix*, Fig. S31*B*).

Samples of OXA-10, treated with tazobactam or tazobactam in combination with BME as described above (with full conversion to the respective −18- and +60-Da species verified by SPE-MS) (*SI Appendix*, Figs. S32 and S33) were crystallized. Three high-resolution structures with two molecules in the asymmetric unit (ASU) were obtained (*SI Appendix*, Table S7), each using similar crystallization conditions (see *Materials and Methods*). In a crystal structure of −18-Da modified OXA-10 derived by tazobactam treatment (PDB: 7B3S, 1.85-Å resolution) clear modification of Ser67 to Dha **9** was observed in both chains, as indicated by the planar geometry of the Cα atom compared to Ser67 of unmodified OXA-10 [PDB: 2X02 (39); Fig. 6 and *SI Appendix*, Fig. S32]. The structure of OXA-10 incubated simultaneously with tazobactam and BME to give a protein with +60-Da mass increment (PDB: 7B3U, 1.60-Å resolution) showed continuous density consistent with Dha-BME **17** formation, both in chain A and chain B (Fig. 6 and *SI Appendix*, Fig. S33). Notably, although generation of small amounts of the *L*(2*R*)-epimer cannot be ruled out, the addition of BME to Dha **9** appears stereoselective with only the *D*(2*S*)-residue being observed. In both chains the hydroxyethyl oxygen is oriented so that the OH is positioned in the same location as the deacylating water in the active site of unmodified OXA-10 (*SI Appendix*, Fig. S33).

In a second structure of the −18-Da modified OXA-10 (PDB: 7B3R, 1.83-Å resolution), connecting electron density was observed between the C-3 side-chain carbon of the original Ser67 and the side-chain nitrogen of Lys70, indicating partial (∼50%) addition of the Lys *N*^ε^-amine to Dha **9**, forming a lysinoalanine (Lal) cross-link **10**; note this species also manifests a −18-Da mass shift (Fig. 6 and *SI Appendix*, Fig. S32). Evidence for cross-linking was observed in both OXA-10 chains. In chain A, clear and continuous electron density for both the Lal cross-link **10** and some unreacted carbamylated lysine was observed. The quality of the electron density map was overall less clear in chain B, though the mF_o_-DF_c_ polder OMIT map (40) indicates partial cross-linking. However, no clear density for the remaining carbamylated lysine was apparent in chain B, which was therefore not included in the final model (*SI Appendix*, Fig. S32*J*). By contrast, with the *D*(2*S*)-stereochemistry observed on BME addition to Dha **9**, the new chiral center formed by reaction of Lys70 with Dha **9** has, at least predominantly, the *L*(2*S*)-stereochemistry.

## Discussion

Removal of the penicillin C-6 side chain coupled with oxidation of the thiazolidine sulfide to a sulfone converts antibiotics into clinically important SBLi, i.e., tazobactam and sulbactam. Enmetazobactam is a new member of the penam sulfone SBLi class, differing from tazobactam solely by addition of a methyl group on its triazole ring, a modification that confers a permanent positive charge, with consequent potential mechanistic effects (20, 33). In accord with prior studies (20, 34), we observed only small differences in potency for enmetazobactam compared to the closely related tazobactam (*SI Appendix*, Tables S1–S3). The reported significantly enhanced potential of enmetazobactam against class A ESBLs in cells (33, 34) and in vivo (41) may thus result from improved properties with respect to variables other than potency against isolated SBLs, e.g., outer membrane permeability or efflux pump susceptibility.

Oxidation of penams to their sulfone state promotes bifurcation of their reaction with SBLs at the acyl–enzyme stage to give transiently inactivating species that are more resistant to hydrolysis than the nascent AEC **2**, turning penam sulfones into transient inactivators of SBLs. Due to the importance of this mechanistic feature for penam sulfone–mediated SBL inhibition, the nature of these transitory stable species has been the subject of extensive kinetic and biophysical studies, including by spectroscopy (6, 7, 9, 10, 12–15), MS (10, 16–22), and crystallography (1, 42).

Several ESI-MS studies under denaturing conditions have implied efficient fragmentation of covalently bound sulfone inhibitors to give the same (+52-, +70-, and +88-Da) fragmented species (**6**, **7**, and **8** in Fig. 2), with no (or little) evidence for species corresponding to the intact inhibitor mass (10, 16–22). By contrast, studies employing Raman (6, 7, 9, 12–15) and UV/Vis (10, 12) spectroscopy and most studies employing crystallography have identified only one transitory stable species, *trans*-enamine **5**, resulting from opening of both β-lactam and thiazolidine rings, but without fragmentation. Our studies on the mechanism of enmetazobactam and related penam sulfones resolve the discrepancies between previous studies employing MS and spectroscopic methods.

By contrast with previous studies employing ESI-MS to investigate SBL inhibition by tazobactam and sulbactam (10, 16–22), SPE-MS studies on the mechanism by which enmetazobactam inhibits representative SBLs indicate that it reacts to give one or more covalently bound species with the mass of the intact inhibitor. Evidence for the reported fragmentations to +52-, +70-, and +88-Da species (corresponding to **6**, **7**, and **8** in Fig. 2) was not observed. The SPE-MS studies, however, did provide evidence for partial fragmentation, to give a previously unidentified (i.e., not reported in studies on sulbactam/tazobactam) +169-Da adduct, potentially resulting from elimination of the triazole and sulfinic acid groups to give alkene **11**. However, studies under native-MS conditions imply that formation of this +169-Da species is promoted under high-energy conditions, generated either by collisional activation in the gas phase for MS analysis or by incubation at higher temperature in the aqueous phase, and thus formation of the +169-Da species is likely of limited relevance in physiological conditions. ESI-MS condition influenced fragmentation of the covalent modifications of SBLs by other SBLi has also been reported, e.g., desulfation of avibactam (43, 44).

Subsequent studies with enmetazobactam, as well as tazobactam and sulbactam, comparing the results obtained by native-, SPE-, and LC-MS, show that the observed lack of +52-, +70-, and +88-Da species in the SPE-MS studies compared to the LC-MS studies with all three penam sulfones results from a milder sample preparation method and does not reflect a distinctive inhibition mode for enmetazobactam. Formation of species with mass shifts corresponding to the reported fragmentation products **6**, **7**, and **8** is promoted by the acidic conditions commonly employed to protonate proteins within conventional denaturing ESI LC-MS workflows (e.g. 0.1% formic acid). For all the penam sulfones, SBL inhibition thus appears to result from formation of transitory stable species with the intact inhibitor mass.

Our combined MS and crystallographic studies (Fig. 4), together with prior crystallographic studies with class A and D enzymes with tazobactam and sulbactam (6–10) and spectroscopic studies (6, 7, 9, 10, 12–15), reveal that the three penam sulfones react similarly, at least on a short timescale under mild/neutral conditions, likely to give predominantly a transitory stable *trans*-enamine sulfuric acid (**5** in Fig. 2).

By contrast with the SPE-MS studies showing evidence for protein-bound species comprising the full mass of the inhibitors (i.e., *trans*-enamine **5** or equivalent mass species) during SBL inhibition, we did not observe evidence for unfragmented AEC-derived hydrolysis products (i.e., enamines/imines) by NMR studies in solution (*SI Appendix*, Figs. S21–S25). Although it is possible that the NMR observed products may (in part) result from fragmentation of enzyme-bound imine/enamine complexes (**3**, **4**, or **5**; Fig. 2) followed by hydrolysis, it is likely that they result from hydrolysis of the nascent acyl–enzyme **2** or imine/enamine intermediates (**3**, **4**, or **5**), followed by efficient fragmentation in solution, as reported for nonenzymatic hydrolysis of clavulanic acid (45, 46).

Although most studies, including our native-MS and SPE-MS studies, point to an AEC species with the full mass of the inhibitor (i.e., *trans*-enamine **5** or equivalent mass species) as the major (but not necessarily sole) species for inhibition, the SPE-MS analysis and some crystallographic (22, 47–49) and UV/Vis-based studies (e.g., ref. 50) provide evidence for rare fragmentation events to give smaller species, e.g. the +169-Da, +52-Da, and −18-Da species. Our results suggest that generation of these species by reaction of SBLs with the current penam sulfones is unlikely to contribute substantially to inhibition in a biological context. However, since some of these species appear resistant to hydrolysis, the future development of sulfone-based SBLi that efficiently generate such species is of interest.

In this regard, it is important to note that by SPE-MS we only observed formation of a +169-Da mass species with enmetazobactam. The additional methyl group of enmetazobactam relative to tazobactam may promote elimination to give the +169-Da species **11**, because it ensures a permanent positive charge on the triazole, thereby promoting its loss. A related fragmentation involving loss of SO_2_ has been observed for 2β-alkenyl penam sulfones with increased activity against class A and C SBLs compared to tazobactam (51). Future studies could thus focus on modification of penam sulfones to promote formation of the +169-Da or equivalent species under biologically relevant conditions.

Most reported evidence suggest a Ser–Ser cross-linked species (i.e., vinyl-ether **8**; Fig. 2) as the molecular basis for the +52-Da species, though there are other possibilities (50, 52, 53). Studies have also shown that the cross-linked +52-Da species may react further to give species with analogous mass increments which confer irreversible inhibition of the SBLs (52). However, in our studies using an excess of tazobactam and enmetazobactam, the small amounts of the +52-Da species that were observed for TEM-116 eventually degraded and did not appear to lead to irreversible inactivation.

A particularly interesting aspect of the prolonged reaction of the penam sulfones with TEM-116 and OXA-10 is formation of catalytically inactive −18-Da species. The combined SPE-MS (including derivatization trials with BME), LC-MS/MS, and crystallographic results imply initial formation of a Dha residue **9**, which reacts with *N*^ε^-amine of a Lys side chain to give the cross-linked Lal species **10** (Fig. 6). Formation of Lal cross-links can occur on treating proteins with heat or high pH and occurs during biosynthesis of protein-derived lanthipeptide antibiotics (54). To date Lal **10** has rarely been identified in natural protein structures under biological conditions, with the spirochaete flagella hook protein being a notable example (55), and it has not previously been described with a β-lactamase. The different stereoselectivities observed for intermolecular (BME) and intramolecular (Lys) additions to Dha **9** are of interest from the perspective of use of Dha in protein engineering (38).

From the inhibition perspective, the Dha **9** and Lal cross-linked **10** species (which cannot be directly distinguished by intact protein MS) are of particular interest as they are the only modifications we observed leading to apparently irreversible inactivation of both class A and D SBLs. While a range of small molecules are known to react with serine, or, more commonly, cysteine residues, to give Dha **9** which may be utilized for protein engineering purposes (38), these compounds usually lack sufficient selectivity and are thus not useful for enzyme inhibition in a biological context. However, selective Dha formation has also been observed on reaction of the class C β-lactamase P99 with β-sultams (56), suggesting general scope for irreversible SBL inhibition through serine dehydration. The apparent differences in efficiency of Dha **9** formation between tazobactam and enmetazobactam on reaction with TEM-116 and OXA-10 shown here (Fig. 5) highlight opportunities for optimization of penam sulfones to result in irreversible β-lactamase inhibition via Dha/Lal formation.

Overall, our work provides insight into the mechanisms of some of the most widely clinically used β-lactamase inhibitors which will help guide their further optimization. The results also further exemplify the power of protein observed MS for studying mechanism-based inhibition involving covalent reactions. However, they show care should be taken in interpretating the MS results, especially when obtained under conditions far from biologically relevant ones, which should be considered in the light of data from other methods.

## Materials and Methods

### Materials.

Inhibitors were from Sigma-Aldrich (tazobactam), Molekula (sulbactam), and MedKoo Biosciences Inc. (enmetazobactam). FC-5 was prepared as reported (35).

### Enzyme Production.

Recombinant AmpC*_EC_*, TEM-116 with an N-terminal His tag, and OXA-10 with a cleavable N-terminal His tag were expressed and purified as previously described (57–59).

### Inhibition Studies.

Kinetic analyses were performed (at least in triplicate) using either BMG LABTECH PHERAstar or CLARIOstar plate readers. Unless stated otherwise, reactions were in 50 mM phosphate buffer, pH 7.5, containing 0.01% (vol/vol) aqueous Triton X-100, and, in the case of OXA-10, 10 mM sodium bicarbonate. Assays were performed in competition with either the fluorescent reporter substrate FC-5 (35), using black-walled microplates (Greiner Bio-One μCLEAR, PS, F-bottom, black), and measuring fluorescence intensity (ʎ_ex_ = 380 nm and ʎ_em_ = 460 nm), or with nitrocefin (NCF), using ultraviolet transparent plates (Greiner Bio-One UV-Star, COC, F-bottom, clear) and measuring absorbance (ʎ_Abs_ = 486 nm, using path-length correction). Nonlinear regression analysis was performed with Prism 5 (GraphPad Software).

IC_50_s were determined as reported (35). TEM-116 (1 nM), AmpC*_EC_* (500 pM), or OXA-10 (250 pM) were incubated with varied inhibitor concentrations at room temperature (r.t.) for the indicated time then assayed using 5 µM FC-5. The apparent inhibitory constant *K*_iapp_ and the second-order rate constant *k*_inact_/*K* (or *k*_2_/*K*) were determined using reported methods (60). SBLs were reacted with the reporter substrate in the presence of varied inhibitor concentrations. Reactions were initiated by SBL addition and immediately monitored for 60 to 120 s (until they plateaued). For TEM-116 (1 nM) assays were carried out in competition with NCF (50 µM); for AmpC*_EC_* (100 nM) and OXA-10 (50 nM) FC-5 (5 µM) was used. *K*_iapp′_ values were obtained by linear regression analysis of initial velocities at varied inhibitor concentrations and corrected to account for the substrate concentration and Michaelis constant (*K*_m_) to give *K*_iapp_. *k*_obs_ values were determined by fitting the obtained progress curves to Eq. **1**, where *P* is formed product, *P*_0_ is background signal, *V_S_* is velocity of no-inhibitor control, *V*_0_ is velocity of no-enzyme control to estimate fully inhibited enzyme, and *t* is time:

[1] $$P=V_{s}t+\left(V_{0}-V_{S}\right)\frac{\left(1-e^{-k_{\text{obs}}t}\right)}{k_{\text{obs}}}+P_{0}.$$

Linear regression of the obtained *k*_obs_ values against the inhibitor concentration gave *k*_inact_/*K′*, which were corrected to account for the substrate concentration and *K*_m_ to give *k*_inact_/*K*.

Dissociation constants (*k*_off_) were determined by the jump dilution method (61). TEM-116 (3 µM) was incubated with the inhibitor (300 µM, 20 min, r.t.). AmpC*_EC_* (3 µM) was incubated with the inhibitor (900 µM, 20 min, r.t.). OXA-10 (3 µM) was incubated with tazobactam (900 µM), enmetazobactam (900 µM), or sulbactam (3 mM) (15 min, r.t.). Reactions were serially diluted (to final concentrations of TEM-116: 30 pM; AmpC*_EC_*: 10 pM, OXA-10: 10 pM) and assayed using 25 µM FC-5. Reactions were monitored for 30 to 400 min then fitted to Eq. **1**, where *V*_S_ is velocity of no-enzyme control to estimate fully inhibited enzyme and *V*_0_ is velocity of no-inhibitor control. Half-lives for SBL inhibition (*t*_1/2_) were obtained using Eq. **2**:

[2] $$t_{1/2}=\;\frac{\text{ln}(2)}{k_{\text{off}}}.$$

The partition ratio (*k*_cat_/*k*_inact_) between transient inhibition and efficient hydrolysis of the AEC was determined as reported (20). TEM-116 (1 nM), AmpC*_EC_* (500 pM), or OXA-10 (250 pM) were incubated with varying concentrations of inhibitors for 60 min at r.t. then assayed with FC-5 (5 µM). The inhibitor–enzyme ratio resulting in ≥90% inhibition was taken as an estimate of the partition ratio.

### High-Performance Liquid Chromatography ESI-MS Assays.

AmpC*_EC_*, OXA-10, or TEM-116 (1 μM) in 50 mM Tris, pH 7.5, were incubated with tazobactam (100 μM), enmetazobactam (100 μM), or sulbactam (500 μM) (15 min, r.t.). Samples were analyzed using a Xevo G2‐S mass spectrometer (Waters) coupled to an Acquity-UPLC system (Waters), equipped with a ProSwift RP-4H 1-mm x 50-mm column (Thermo Fisher Scientific), loaded onto the column in 95% (vol/vol) water, 5% (vol/vol) acetonitrile, and 0.1% (vol/vol) formic acid, then eluted using a gradient to 5% (vol/vol) water, 95% (vol/vol) acetonitrile, and 0.1% (vol/vol) formic acid over 10 min, then introduced directly into the ESI source. Retention times of all proteins were ∼4 to 5 min. Data were analyzed using MassLynx 4.1 (Waters), with deconvolution using the MaxEnt1 algorithm.

### Solid-Phase Extraction (SPE) ESI-MS Assays.

AmpC*_EC_*, OXA-10, or TEM-116 (3 μM) in 50 mM Tris, pH 7.5, were incubated with tazobactam (30 μM or 300 μM), enmetazobactam (30 μM or 300 μM), or sulbactam (300 μM or 1.5 mM) (r.t.). Mass spectra were acquired in the positive ion mode using an integrated autosampler/SPE RapidFire365 system (Agilent Technologies) coupled to an Agilent 6550 Accurate Mass QTOF spectrometer. After the indicated time, 50 μL of the solution was loaded onto a C4 SPE cartridge (Agilent Technologies), then washed with buffer A (100% [vol/vol] water, 0.1% [vol/vol] formic acid), then eluted into the mass spectrometer in buffer B (15% [vol/vol] water, 85% [vol/vol] acetonitrile, 0.1% [vol/vol] formic acid). The cartridge was reequilibrated in buffer A in between samples. Data were analyzed using MassHunter Qualitative Analysis software V.7 (Agilent Technologies) using the maximum entropy deconvolution algorithm.

### Nondenaturing MS Assays.

AmpC*_EC_*, OXA-10, or TEM-116 (10 μM) were incubated with tazobactam (200 μM) or enmetazobactam (200 μM) in 1 M ammonium acetate, pH 7.5, or 50 mM Tris, pH 7.5. Samples were exchanged into 1 M ammonium acetate solution, pH 7.5, using Zeba micro spin desalting columns (7,000 molecular weight cutoff [MWCO]; Thermo Fisher Scientific) as described by the manufacturer. Samples (3 µL) were loaded into in-house prepared gold-coated capillary needles (Harvard Apparatus) and were injected into a Q-Exactive UHMR Hybrid Quadrupole-Orbitrap spectrometer (Thermo Fisher Scientific) (62). Instrument parameters: capillary voltage 1.2 kV, S-lens RF 200%, mass range from 1,000 to 12,000 *m/z*, capillary temperature 60 °C, resolution of the instrument 17,500 at *m/z* = 200 (transient time: 64 ms). The noise level was 3, rather than the default of 4.64. In some cases in-source dissociation energy (0 to 100 V) was applied. While analyzing the dependence of elimination from the AmpC*_EC_*–enmetazobactam-derived complex, the HCD activation collisional energy and UHV gas pressure were varied. Calibration of the instruments was performed using a 10 mg⋅mL^−1^ solution of CsI in water. Data were analyzed using Xcalibur 4.1 (Thermo Fisher Scientific).

### Trypsin Digestions and MS/MS Analyses.

OXA-10 and TEM-116 (3 μM) were incubated overnight with tazobactam (300 μM) or enmetazobactam (300 μM), then analyzed by SPE-MS. Samples were exchanged into 50 mM NaHCO_3_ buffer and concentrated to 8 µM using an Amicon Ultra centrifugal filter (10,000 MWCO; Merck Millipore). Proteins were denatured at 95 °C for 10 min, then cooled to r.t. After addition of 4 mM dithiothreitol (DTT), samples were incubated (25 min, 56 °C, shaking), cooled to r.t. before addition of 8 mM iodoacetamide, and incubated (30 min, r.t., in the dark). Residual iodoacetamide was quenched by addition of 4 mM DTT before addition of trypsin (160 nM, 1:50). Samples were incubated (overnight, 37 °C) then quenched with 10% (vol/vol) aqueous formic acid solution.

Peptides were analyzed using an UltiMate 3000 UHPLC connected to an Orbitrap Eclipse Tribrid spectrometer (Thermo Fisher Scientific). They were trapped on a guard column (Acclaim PepMap 100, 75 µm × 2 cm, nanoViper, C18, 3 µm, 100 Å; Thermo Fisher Scientific) using solvent A (0.1% [vol/vol] aqueous formic acid), then separated on an Acclaim PepMap analytical column (75 µm × 150 mm, RSLC C18, 3 µm, 100 Å; Thermo Fisher Scientific) using a linear gradient (6 to 45% solvent B [0.1% formic acid, 80% acetonitrile, 20% water], 300 nL/min, 90 min). The separated peptides were electrosprayed directly into the mass spectrometer (positive ion mode/data-dependent acquisition with a 3 s cycle time). Precursors and products were detected at a resolving power of 60,000 and 30,000 (at *m/z* 200), respectively. Precursor signals with an intensity >1.0 × 10^−4^ and charge state between 2 and 7 were isolated with the quadrupole using a 0.7 *m/z* isolation window (0.5 *m/z* offset) and subjected to MS/MS fragmentation using higher-energy collision-induced dissociation (30% relative fragmentation energy). MS/MS scans were collected at an AGC setting of 1.0 × 10^4^ or a maximum fill time of 100 ms and precursors within 10 ppm were dynamically excluded for 30 s.

Raw data files were processed using MaxQuant Version 1.6.3.4 with the Andromeda search engine (63, 64). Peak lists were searched against individual sequences and potential contaminant proteins. Carbamidomethylation was kept as a fixed modification whereas acetylation (protein N-term), oxidation (methionine), and dehydration (serine) and its corresponding adducts with BME and DTT were variable modifications. The peptide false discovery rate was kept at 1%. Trypsin was set as the protease and up to four missed cleavages were allowed. Spectra identifying cross-linked peptides were manually validated.

### Crystallography.

Crystallization was by vapor diffusion at r.t. employing a Rigaku Phoenix RE Drop setter instrument and low reservoir Intelli-Plates 93-3 (Art Robbins). Unmodified AmpC*_EC_* crystallized as described (59). Crystals were transferred into precipitant solution supplemented with enmetazobactam (200 mM), incubated (14 min, r.t.), then cryocooled and stored in liquid nitrogen.

OXA-10 (3 µM in 50 mM Tris, pH 7.5) was incubated with tazobactam (300 µM) for 18 h at r.t., with rocking. Apparently complete modification of OXA-10 to give the –18-Da species was verified by SPE-MS. Prior to crystallization the sample was concentrated to a final OXA-10 concentration of 10 mg⋅mL^−1^. Screening the Pact Premier suite (Molecular Dimensions) gave crystals in condition G8 (0.2 M sodium sulfate, 0.1 M Bis-Tris propane, pH 7.5, 20% [wt/vol] PEG 3350) showing OXA-10 S70Dha modification (PDB: 7B3S) and condition F5 (0.2 M sodium nitrate, 0.1 M Bis-Tris propane, pH 6.5, 20% [wt/vol] PEG 3350) showing OXA-10 67-70Lal (PDB: 7B3R). Crystals appeared within 24 h and were cryocooled and stored in liquid nitrogen.

Crystals of the OXA-10-BME species (PDB: 7B3U) were obtained by incubating OXA-10 (3 µM in 50 mM Tris, pH 7.5) with tazobactam (300 µM) and BME (3 mM) for 18 h at r.t., with rocking. Apparent complete modification of OXA-10 to give the +60-Da species was observed by SPE-MS; small molecules were removed using an Amicon concentrator (10,000 MWCO; Merck Millipore). The sample was washed with 50 mM Tris, pH 7.5 (3 × 10 mL) then concentrated to 10 mg⋅mL^−1^. Screening the Pact Premier suite (Molecular Dimensions) gave crystals in condition F8 (0.2 M sodium sulfate, 0.1 M Bis-Tris propane, pH 6.5, 20% [wt/vol] PEG 3350). Crystals appeared within 24 h and were cryocooled and stored in liquid nitrogen.

Data were collected at beamline i03 at the Diamond Light Source (*SI Appendix*, Table S3) and were indexed, integrated, and scaled using Xia2 (65). Structures were solved by molecular replacement using Phaser (66) [using 6T3D (59) (AmpC*_EC_*) and 1K55 (36) (OXA-10)]. Alternating cycles of refinement using PHENIX (67) and model building using Coot (68) were performed until *R*_work_ and *R*_free_ converged.

### Nuclear Magnetic Resonance (NMR) Spectroscopy.

^1^H NMR spectra were recorded using a Bruker BioSpin Avance 750 MHz spectrometer and a 5-mm TCI cryoprobe. The buffer was 50 mM sodium phosphate, pH 7.5, and 10% (vol/vol) D_2_O. The water signal was suppressed using presaturation or excitation sculpting with the Perfect Echo pulse sequence (69). Assays were performed using 1 or 5 mM enmetazobactam, tazobactam, or sulbactam and 10 μM AmpC*_EC_*, TEM-116, or OXA-10. Chemical shift assignments were made on the basis of ^1^H, correlation (COSY), heteronuclear single quantum coherence (HSQC), and heteronuclear multiple bond connectivity (HMBC) spectra and a previous report (70).

## Supplementary Material

Supplementary File

## Data Availability

Protein crystal structures have been deposited in the PDB (6T35, 7B3S, 7B3R, and 7B3U).
