# Supplementary material for: Studies on enmetazobactam clarify mechanisms of widely used β-lactamase inhibitors
Source: Proc Natl Acad Sci U S A. 2022 Apr 29;119(18):e2117310119. doi: 10.1073/pnas.2117310119 (PMC9170034; doi:10.1073/pnas.2117310119)
Supplement: Supplementary File [file pnas.2117310119.sapp.pdf]

Supplementary Information

Studies on enmetazobactam clarify mechanisms of widely used β-lactamase inhibitors

Pauline A. Lang[1], Ritu Raj[2,3], Anthony Tumber[1], Christopher T. Lohans[1,4], Patrick Rabe[1], Carol V. Robinson[2,3], Jürgen Brem[1,5]*, and Christopher J. Schofield[1]*

[1] Chemistry Research Laboratory, Department of Chemistry and the Ineos Oxford Institute for Antimicrobial Research, University of Oxford, 12 Mansfield Road, Oxford, OX1 3TA, United Kingdom.

[2] Physical and Theoretical Chemistry Laboratory, Department of Chemistry, University of Oxford, Oxford, OX1 3TA, United Kingdom.

[3] The Kavli Institute for Nanoscience Discovery, University of Oxford, Oxford, OX1 3QU, United Kingdom.

[4] Current address: Department of Biomedical and Molecular Sciences, Queen's University, Kingston, K7L 3N6, Canada.

[5] Current address: Janssen Research & Development, Janssen Pharmaceutica, B-2340 Beerse, Belgium.

*Correspondence should be addressed to Jürgen Brem and Christopher J. Schofield.

Email: bremjurgen@yahoo.com and christopher.schofield@chem.ox.ac.uk

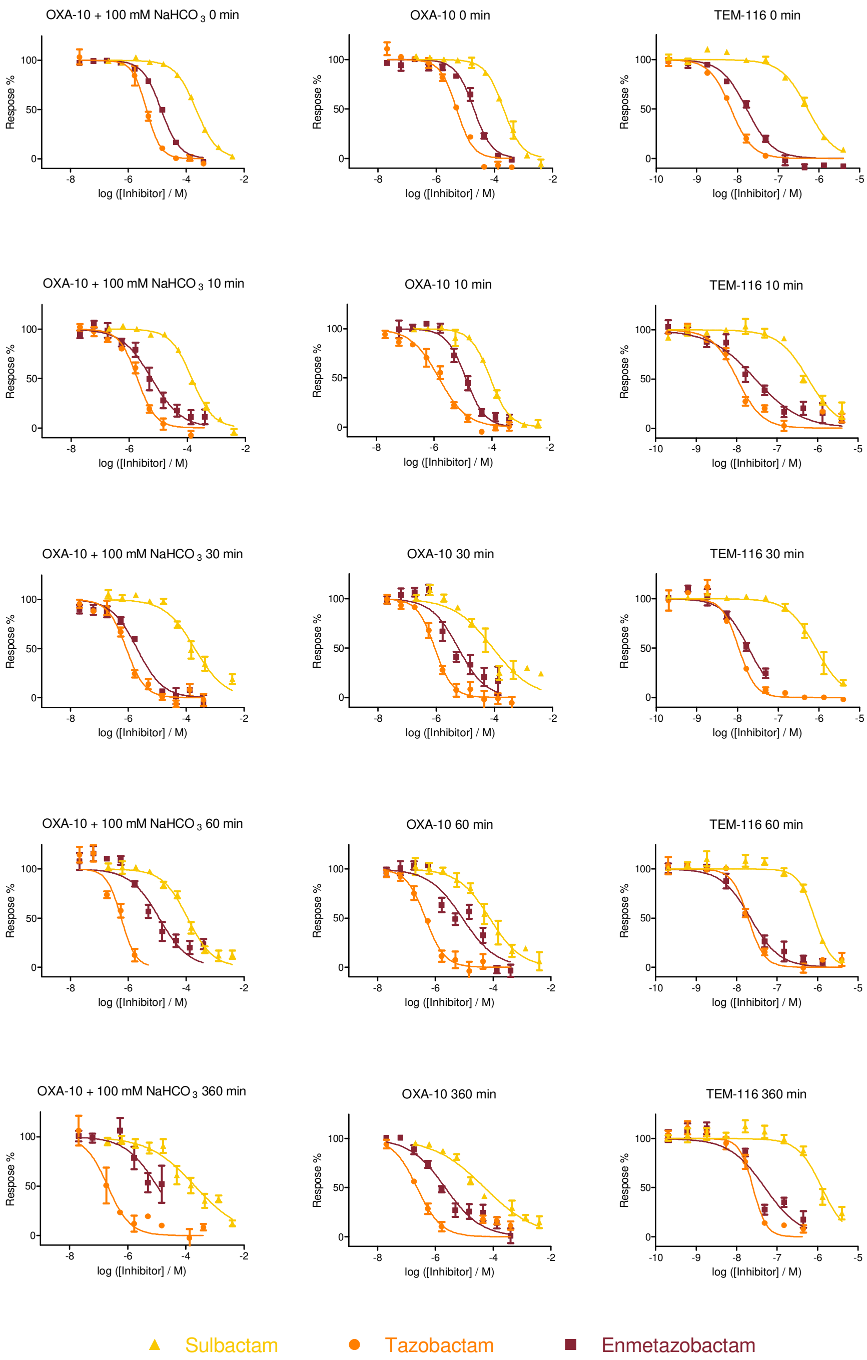

Figure S1: Dose dependence curves for OXA-10 and TEM-116 inhibition by tazobactam, enmetazobactam, or sulbactam. Enzymes (1 nM TEM-116 or 250 pM OXA-10) in 50 mM phosphate buffer pH 7.5, 0.01% (v/v) Triton X-100 were pre-incubated with inhibitors for the indicated times at r.t., then assayed using 5 µM FC-5(1). OXA-10 was also screened with 100 mM $NaHCO_3$. pIC_{50}s were determined using non-linear regression (Table S1). Error bars indicate standard errors of four technical replicates.

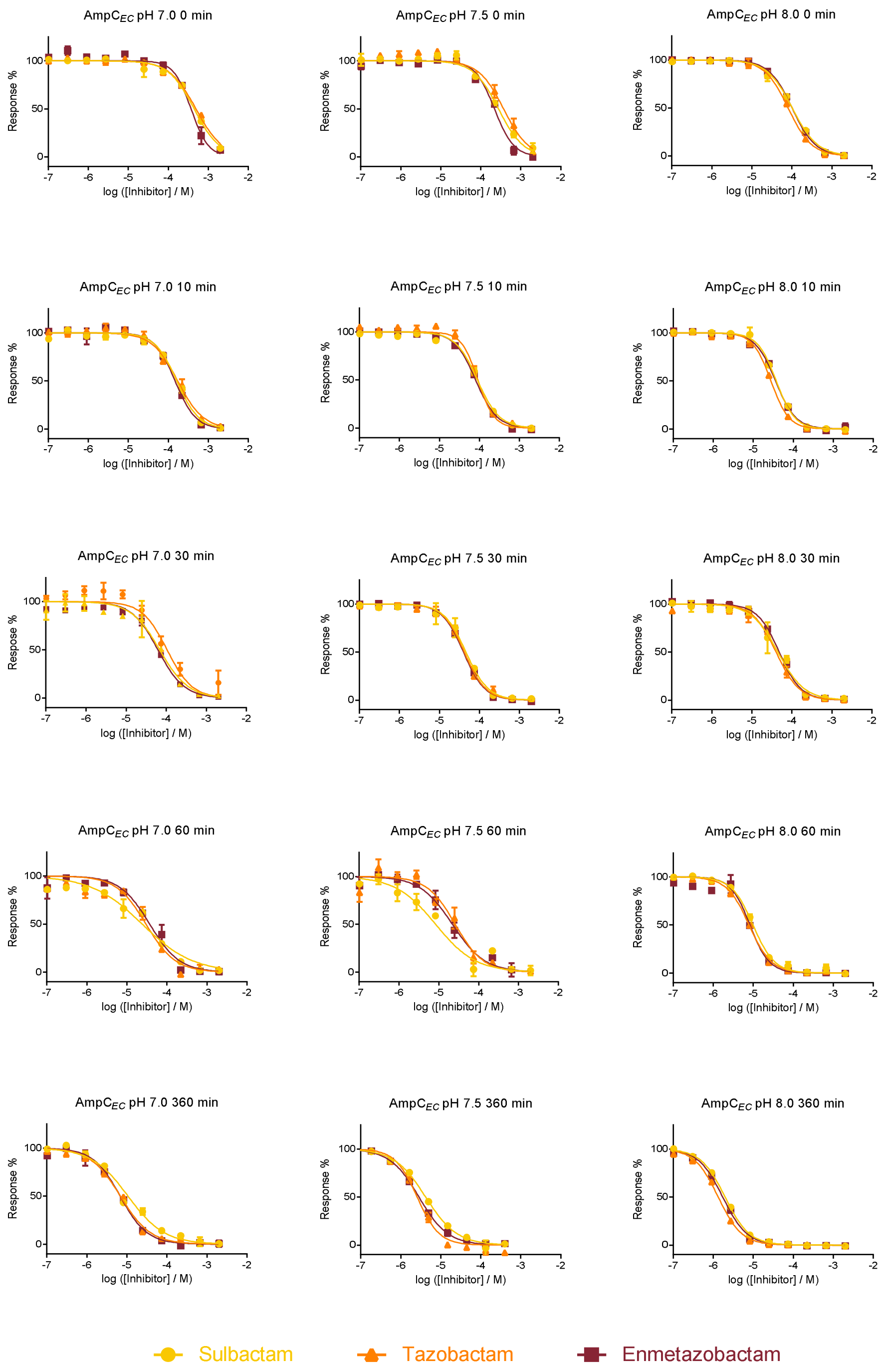

Figure S2: Dose dependence curves for AmpC$_{EC}$ inhibition by tazobactam, enmetazobactam, or sulbactam. AmpC$_{EC}$ (500 pM) was pre-incubated with the inhibitors for the indicated times at r.t. and assayed using 5 µM FC-5(1). Buffer: 50 mM phosphate buffer, pH 7.0, 7.5, or 8.0 and 0.01% (v/v) Triton X-100. pIC$_{50}$s were determined using non-linear regression (Table S1 and S2). Error bars indicate standard errors of four technical replicates.

Table S1: Time dependence of TEM-116, AmpC*EC*, and OXA-10 and inhibition by tazobactam, enmetazobactam, or sulbactam. Enzymes (1 nM TEM-116, 500 pM AmpC*EC* or 250 pM OXA-10) in 50 mM phosphate buffer pH 7.5, 0.01% (v/v) Triton X-100 were pre-incubated with inhibitors for the indicated times at r.t., then assayed using 5 µM FC-5(1). OXA-10 was also screened in the presence of added 100 mM $NaHCO_3$. pIC_{50}s were determined using non-linear regression (Figure S1). Errors are standard errors of four technical replicates.

		pIC_{50}[a] at pH 7.5			
	time	OXA-10[a]	OXA-10[b]	TEM-116	AmpC*EC*
tazobactam	0 min	5.41 ± 0.07	5.30 ± 0.04	8.16 ± 0.05	3.43 ± 0.04
	10 min	5.72 ± 0.05	5.83 ± 0.06	7.98 ± 0.07	4.04 ± 0.02
	30 min	6.07 ± 0.05	6.05 ± 0.06	7.97 ± 0.03	4.52 ± 0.02
	60 min	6.25 ± 0.07	6.34 ± 0.07	7.68 ± 0.07	4.79 ± 0.02
	360 min	6.69 ± 0.12	6.63 ± 0.08	7.60 ± 0.04	5.59 ± 0.03
enmetazobactam	0 min	4.86 ± 0.02	4.71 ± 0.04	7.77 ± 0.04	3.66 ± 0.02
	10 min	5.23 ± 0.08	4.90 ± 0.05	7.51 ± 0.12	4.10 ± 0.02
	30 min	5.70 ± 0.08	5.24 ± 0.10	7.73 ± 0.05	4.55 ± 0.03
	60 min	4.75 ± 0.14	4.97 ± 0.14	7.65 ± 0.07	4.81 ± 0.01
	360 min	4.99 ± 0.19	5.49 ± 0.11	7.28 ± 0.09	5.55 ± 0.02
sulbactam	0 min	3.66 ± 0.01	3.69 ± 0.04	6.27 ± 0.03	3.56 ± 0.03
	10 min	3.85 ± 0.02	4.06 ± 0.03	6.23 ± 0.05	4.08 ± 0.05
	30 min	3.58 ± 0.08	4.05 ± 0.10	6.08 ± 0.04	4.44 ± 0.03
	60 min	3.95 ± 0.05	4.12 ± 0.08	6.07 ± 0.04	4.70 ± 0.02
	360 min	3.69 ± 0.10	4.30 ± 0.08	5.89 ± 0.06	5.38 ± 0.03

[a]With100 mM added $NaHCO_3$.

[b]Without added $NaHCO_3$.

Table S2: pH and time dependence of AmpC*EC* inhibition by tazobactam, enmetazobactam, or sulbactam. AmpC*EC* (500 pM) was pre-incubated with the inhibitors for the indicated times at r.t., then assayed using 5 µM FC-5(1). Buffer: 50 mM phosphate buffer, pH 7.0, 7.5, or 8.0 and 0.01% (v/v) Triton X-100. pIC_{50}s were determined using non-linear regression (Figure S2). Errors are standard errors of four technical replicates.

			pIC_{50}	
	time	pH 7.0	pH 7.5	pH 8.0
tazobactam	0 min	3.31 ± 0.03	3.43 ± 0.04	4.09 ± 0.02
	10 min	3.77 ± 0.03	4.04 ± 0.02	4.56 ± 0.02
	30 min	4.01 ± 0.04	4.52 ± 0.02	N/D
	60 min	4.38 ± 0.03	4.79 ± 0.02	5.04 ± 0.04
	360 min	5.16 ± 0.03	5.59 ± 0.03	5.88 ± 0.01
enmetazobactam	0 min	3.43 ± 0.03	3.66 ± 0.02	3.99 ± 0.01
	10 min	3.83 ± 0.02	4.10 ± 0.02	4.44 ± 0.02
	30 min	4.20 ± 0.03	4.55 ± 0.03	N/D
	60 min	4.40 ± 0.02	4.81 ± 0.01	5.08 ± 0.05
	360 min	5.17 ± 0.03	5.55 ± 0.02	5.76 ± 0.01
sulbactam	0 min	3.35 ± 0.02	3.56 ± 0.03	4.01 ± 0.04
	10 min	3.79 ± 0.02	4.08 ± 0.05	4.43 ± 0.02
	30 min	4.16 ± 0.07	4.44 ± 0.03	N/D
	60 min	4.34 ± 0.04	4.70 ± 0.02	5.01 ± 0.02
	360 min	4.94 ± 0.04	5.38 ± 0.03	5.77 ± 0.03

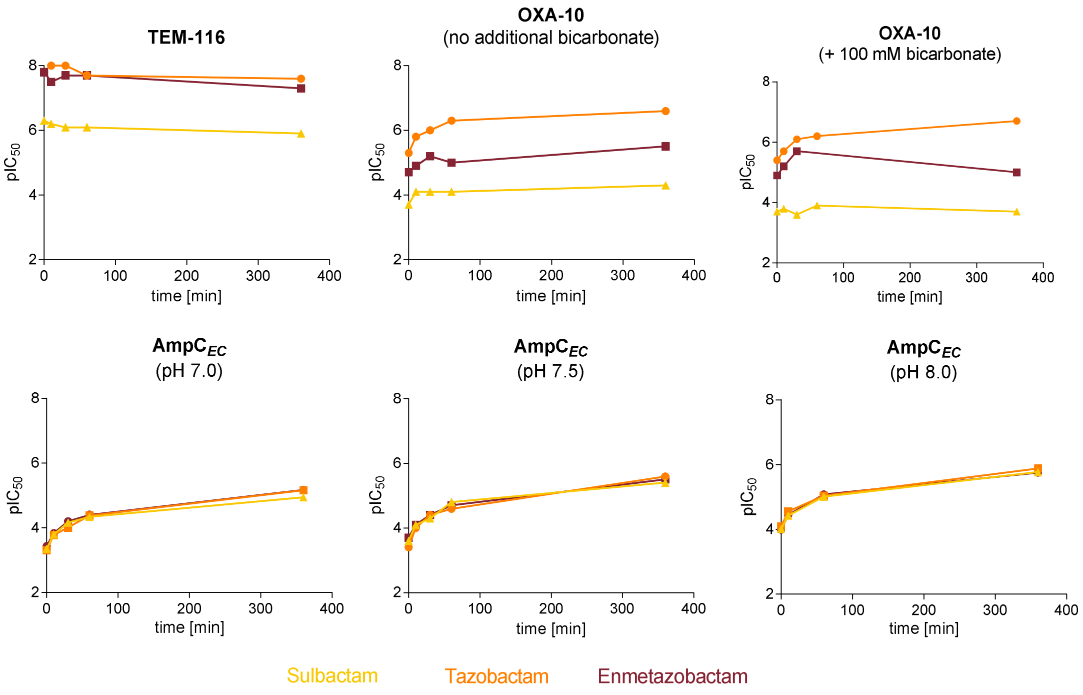

Figure S3: Time dependence of TEM-116, AmpC$_{EC}$, and OXA-10 inhibition by tazobactam, enmetazobactam, or sulbactam. The enzymes (1 nM TEM-116, 500 pM AmpC$_{EC}$ or 250 pM OXA-10) in 50 mM phosphate buffer pH 7.5, 0.01% (v/v) Triton X-100 were pre-incubated with inhibitors for the indicated times at r.t., then assayed using 5 μM FC-5(1). OXA-10 was also screened with 100 mM $NaHCO_3$ added. pIC$_{50}$s were determined using non-linear regression (Figure S1 and S2), as summarized in Tables S1-2.

Table S3: Inhibition of TEM-116, AmpC$_{EC}$, and OXA-10 by enmetazobactam, tazobactam, or sulbactam compared to reported values for other serine β-lactamases. IC_{50}s in bold are from the current work and were determined in assays using FC-5 (5 µM)(1). The SBLs (1 nM TEM-116, 500 pM AmpC$_{EC}$ or 250 pM OXA-10) in 50 mM phosphate buffer pH 7.5, 0.01 % (v/v) Triton X-100 were pre-incubated with inhibitors (10 min, r.t.). With OXA-10, 100 mM $NaHCO_3$ was added. In parentheses are pIC_{50}s with standard errors of four technical replicates. Reported IC_{50}s (not in bold) employed periplasmic extracts containing SBLs and used nitrocefin as a reporter substrate (5 min pre-incubation).(2) ND: Not determined.

	**IC_{50} (µM)**		
	enmetazobactam	tazobactam	sulbactam
**Class A**			
**TEM-116**	**0.036 (7.51 ± 0.12)**	**0.011 (7.98 ± 0.07)**	**0.59 (6.23 ± 0.05)**
TEM-1(2)	0.007 ± 0.001	0.006 ± 0.001	ND
TEM-26(2)	0.008 ± 0.009	0.023 ± 0.001	ND
TEM-30(2)	0.29 ± 0.03	0.24 ± 0.003	ND
SHV-1(2)	0.008 ± 0.001	0.026 ± 0.003	ND
SHV (R244S)(2)	0.56 ± 0.06	0.39 ± 0.04	ND
SHV-49(2)	0.36 ± 0.04	0.73 ± 0.08	ND
CTX-M-14(2)	0.006 ± 0.001	0.010 ± 0.001	ND
CTX-M-15(2)	0.007 ± 0.001	0.006 ± 0.001	ND
KPC-2(2)	0.36 ± 0.04	3.7 ± 0.4	ND
KPC-3(2)	0.52 ± 0.05	4.4 ± 0.5	ND
**Class C**			
**AmpC *E. coli***	**80.82 (4.10 ± 0.02)**	**91.70 (4.04 ± 0.02)**	**89.19 (4.08 ± 0.05)**
AmpC *E. cloacae(2)*	25 ± 3	5.1 ± 0.5	ND
CMY-2(2)	31 ± 3	2.5 ± 0.3	ND
**Class D**			
**OXA-10**	**5.9 (4.90 ± 0.05)**	**1.9 (5.83 ± 0.06)**	**142.5 (4.06 ± 0.03)**
OXA-1(2)	8.7 ± 0.9	8.6 ± 0.9	ND
OXA-48(2)	11 ± 1	1.5 ± 0.2	ND
OXA-51(2)	> 100	> 100	ND
OXA-58(2)	> 100	26 ± 3	ND

A

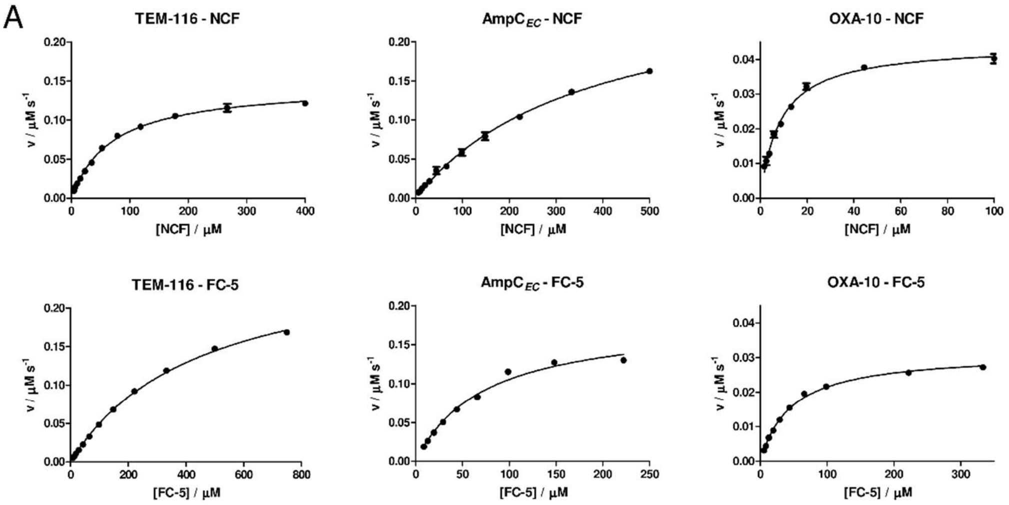

B

	k_{cat} (s^{-1})	K_m (µM)	k_{cat}/K_m (µM^{-1} s^{-1})
**TEM-116**			
NCF	145.8 ± 2.9	70.7 ± 3.1	2.06 ± 0.12
FC-5	274.1 ± 3.6	448.8 ± 11.2	0.611 ± 0.023
**AmpC$_{EC}$**			
NCF	563.3 ± 27.6	367.5 ± 30.8	1.53 ± 0.20
FC-5	366.2 ± 10.9	73.6 ± 5.0	4.97 ± 0.49
**OXA-10**			
NCF	177.0 ± 3.6	8.5 ± 0.5	20.7 ± 1.7
FC-5	126.0 ± 2.4	46.4 ± 2.3	2.72 ± 0.18

Figure S4: Kinetic characterization of SBL catalyzed hydrolysis of reporter substrates FC-5 and Nitrocefin (NCF). TEM-116 (1 nM), AmpCEC (500 pM), or OXA-10 (250 pM) were reacted with varying concentrations of NCF or FC-5. Buffer: 50 mM phosphate buffer, pH 7.5, 0.01% (v/v) Triton X-100. For experiments with OXA-10 the buffer additionally contained 10 mM $NaHCO_3$.

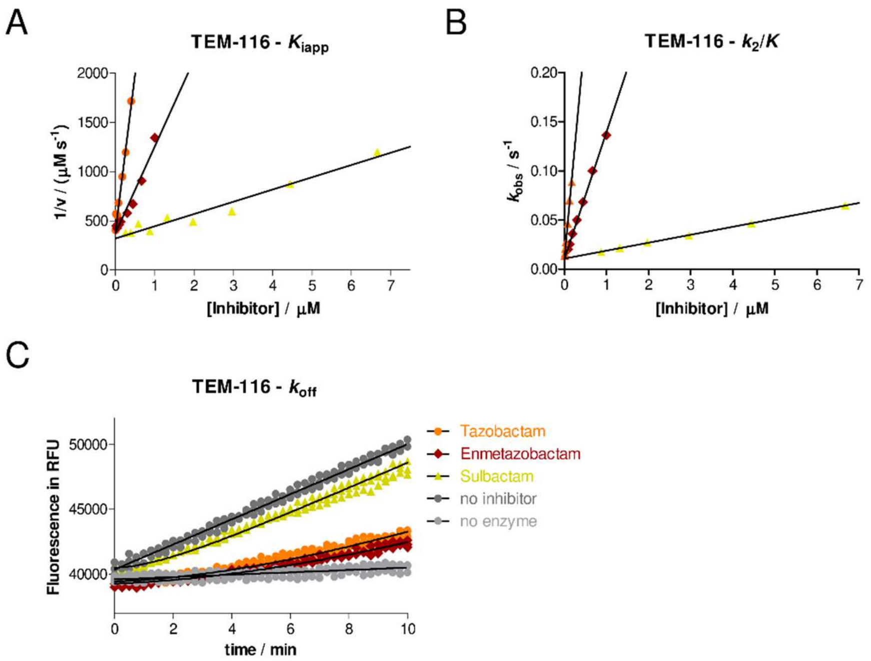

Figure S5: Kinetic analyses of TEM-116 inhibition by penam sulfones. (A) Determination of K_{iapp} values for TEM-116 (1 nM) inhibition, assayed with Nitrocefin (50 µM). (B) Pseudo first-order rate (k_{inact}/K) determination for TEM-116 (1 nM), assayed with Nitrocefin (50 µM). (C) Dissociation after jump-dilution of TEM-116 (3 µM) pre-incubated with tazobactam, enmetazobactam, or sulbactam (300 µM) at r.t. for 20 min, then diluted 100,000 fold and assayed with FC-5(1) (25 µM). Buffer: 50 mM phosphate buffer, pH 7.5, containing 0.01 % (v/v) Triton-X 100.

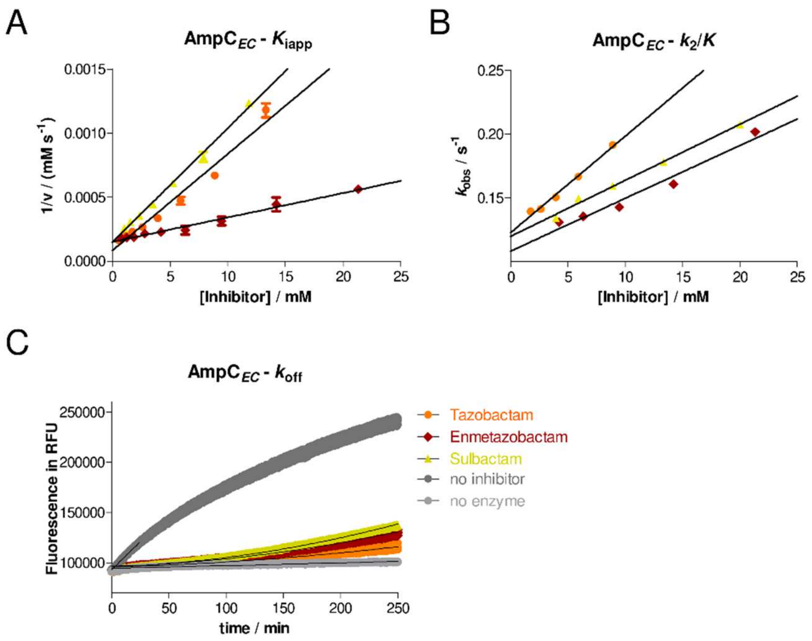

Figure S6: Kinetic analyses of AmpC*EC* inhibition by penam sulfones. (A) Determination of K_{iapp} values for AmpC*EC* (100 nM) inhibition, assayed with FC-5(1) (5 µM). (B) Pseudo first-order rate (k_{inact}/K) determination for AmpC*EC* (100 nM), assayed with FC-5 (5 µM). (C) Dissociation after jump-dilution of AmpC*EC* (3 µM) pre-incubated with tazobactam, enmetazobactam, or sulbactam (900 µM) at r.t. for 20 min, then diluted 300,000 fold and assayed with FC-5(1) (25 µM). Buffer: 50 mM phosphate buffer, pH 7.5, containing 0.01 % (v/v) Triton-X 100.

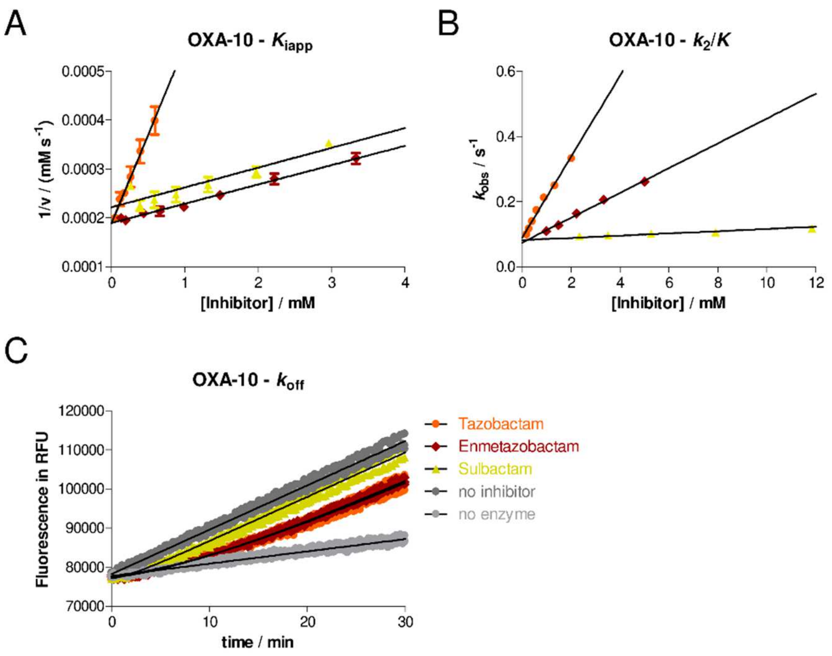

Figure S7: Kinetic analyses of OXA-10 inhibition by penam sulfones. (A) Determination of K_{iapp} values for OXA-10 (50 nM) inhibition, assayed with FC-5(1) (5 µM). (B) Pseudo first-order rate (k_{inact}/K) determination for OXA-10 (50 nM), assayed with FC-5 (5 µM). (C) Dissociation after jump-dilution of OXA-10 (3 µM) pre-incubated with tazobactam (900 µM), enmetazobactam (900 µM), or sulbactam (3 mM) at r.t. for 15 min, then diluted 300,000 fold and assayed with FC-5(1) (25 µM). Buffer: 50 mM phosphate buffer, pH 7.5, containing 0.01 % (v/v) Triton-X 100 and 10 mM $NaHCO_3$.

Table S4: Kinetic parameters for transient SBL inhibition by penam sulfones. Values as determined from regressions in Figure S5-7. Buffer: 50 mM phosphate buffer, pH 7.5, 0.01% Triton X-100. For experiments with OXA-10 the buffer additionally contained 10 mM $NaHCO_3$.

	K_{iapp} (µM)	k_{inact}/K (M^{-1} s^{-1})	k_{off} (M^{-1} s^{-1})	$t_{1/2}$ (min)	k_{cat}/k_{inact}
**TEM-116**					
Tazobactam	0.07 ± 0.01	(746 ± 32) x 10^3	(1.47 ± 0.04) x 10^{-3}	7.8 ± 0.2	9
Enmetazobactam	0.26 ± 0.04	(209 ± 5) x 10^3	(1.06 ± 0.03) x 10^{-3}	10.9 ± 0.3	4
Sulbactam	1.59 ± 0.28	(13.1 ± 0.1) x 10^3	(9.26 ± 0.56) x 10^{-3}	1.2 ± 0.1	240
**AmpC*EC***					
Tazobactam	1042 ± 242	7.6 ± 0.6	(6.62 ± 0.4) x 10^{-6}	1746 ± 10	9800
Enmetazobactam	7464 ± 985	4.1 ± 0.5	(14.4 ± 0.4) x 10^{-6}	803 ± 2	5100
Sulbactam	1577 ± 189	4.9 ± 0.3	(18.8 ± 0.4) x 10^{-6}	616 ± 1	3100
**OXA-10**					
Tazobactam	469 ± 80	138 ± 7	(1.21 ± 0.02) x 10^{-3}	9.6 ± 0.2	160
Enmetazobactam	4311 ± 365	42 ± 2	(1.36 ± 0.02) x 10^{-3}	8.5 ± 0.1	1700
Sulbactam	4916 ± 873	3.8 ± 0.1	(7.46 ± 0.75) x 10^{-3}	1.6 ± 0.2	53000

Table S5: Modifications to AmpC$_{EC}$, TEM-116 and OXA-10 by reaction with enmetzobactam, tazobactam, or sulbactam as analyzed by liquid chromatography (LC) MS, solid phase extraction (SPE) MS and non-denaturing (native) MS techniques. Differences between measured and calculated masses / mass shifts are likely within experimental error.

		Mass observed[a] [Da]	Mass calculated [Da]	Mass shift observed[b] [Da]	Mass shift calculated [Da]	Assignment[c]	Comments
**LC-MS**	**AmpC$_{EC}$**	39552	39551			unmodified **1**	
(4 min	+enmetazobactam	39603	39603	+51	+52	crosslink **8**	or equal mass species
exposure to		39621	39621	+69	+70	aldehyde **7**	
0.1 %		39640	39639	+88	+88	hydrated aldehyde **6**	
formic acid)	+ tazobactam	39603	39603	+51	+52	crosslink **8**	or equal mass species
		39621	39621	+69	+70	aldehyde **7**	
		39640	39639	+88	+88	hydrated aldehyde **6**	
	+ sulbactam	39602	39603	+50	+52	crosslink **8**	or equal mass species
		39621	39621	+69	+70	aldehyde **7**	
		39640	39639	+88	+88	hydrated aldehyde **6**	
	**OXA-10**	27681	27682			unmodified **1**	
	+enmetazobactam	27732	27734	+51	+52	crosslink **8**	or equal mass species
		27750	27752	+69	+70	aldehyde **7**	
		27769	27770	+88	+88	hydrated aldehyde **6**	
	+ tazobactam	27732	27734	+51	+52	crosslink **8**	or equal mass species
		27750	27752	+69	+70	aldehyde **7**	
		27768	27770	+87	+88	hydrated aldehyde **6**	
	+ sulbactam	27732	27734	+51	+52	crosslink **8**	or equal mass species
		27750	27752	+69	+70	aldehyde **7**	
		27768	27770	+87	+88	hydrated aldehyde **6**	
	**TEM-116**	32104	32103			unmodified **1**	
	+enmetazobactam	32156	32155	+52	+52	crosslink **8**	or equal mass species
		32175	32173	+71	+70	aldehyde **7**	
		32193	32191	+89	+88	hydrated aldehyde **6**	
	+ tazobactam	32156	32155	+52	+52	crosslink **8**	or equal mass species
		32175	32173	+71	+70	aldehyde **7**	
		32193	32191	+89	+88	hydrated aldehyde **6**	
	+ sulbactam	32156	32155	+52	+52	crosslink **8**	or equal mass species
		32175	32173	+71	+70	aldehyde **7**	
		32193	32191	+89	+88	hydrated aldehyde **6**	
**SPE-MS**	**AmpC$_{EC}$**	39551	39551			unmodified **1**	
(4 s	+ enmetazobactam	39718	39720	+167	+169	elimination product **11**	formation induced > 55 °C
exposure to		39865	39865	+314	+314	imine/enamine **3, 4, or 5**	or equal mass species (**2**)
0.1 %	+ tazobactam	39851	39851	+300	+300	imine/enamine **3, 4, or 5**	or equal mass species (**2**)
formic acid)	+ sulbactam	39784	39784	+233	+233	imine/enamine **3, 4, or 5**	or equal mass species (**2**)
	**OXA-10**	27681	27682			unmodified **1**	
	+ enmetazobactam	27847	27851	+166	+169	elimination product **11**	formation induced > 55 °C
		27995	27996	+314	+314	imine/enamine **3, 4, or 5**	or equal mass species (**2**)
		27662	27662	-19	-18	dehydroalanine **9**	or lysinoalanine **10**
	+ tazobactam	27981	27982	+300	+300	imine/enamine **3, 4, or 5**	or equal mass species (**2**)
		27662	27963	-19	-19	dehydroalanine **9**	or lysinoalanine **10**
	+ sulbactam	27913	27915	+232	+233	imine/enamine **3, 4, or 5**	or equal mass species (**2**)
		27662	27964	-19	-18	dehydroalanine **9**	or lysinoalanine **10**
	**TEM-116**	32103	32103			unmodified **1**	
	+enmetazobactam	32270	32272	+167	+169	elimination product **11**	formation induced > 55 °C
		32417	32417	+314	+314	imine/enamine **3, 4, or 5**	or equal mass species (**2**)
		32154	32155	+51	+52	crosslink **8**	or equal mass species
		32085	32085	-18	-18	dehydroalanine **9**	or lysinoalanine **10**
	+ tazobactam	32404	32403	+301	+300	imine/enamine **3, 4, or 5**	or equal mass species (**2**)
		32154	32155	+51	+52	crosslink **8**	or equal mass species
		32081	32085	-22	-18	dehydroalanine **9**	or lysinoalanine **10**
	+ sulbactam	32336	32336	+233	+233	imine/enamine **3, 4, or 5**	or equal mass species (**2**)
**Native-MS**	**AmpC$_{EC}$**	39551	39551			unmodified **1**	
(no	+ enmetazobactam	39866	39865	+315	+314	imine/enamine **3, 4, or 5**	or equal mass species (**2**)
exposure to	+ tazobactam	39851	39851	+300	+300	imine/enamine **3, 4, or 5**	or equal mass species (**2**)
formic acid)	**OXA-10**	55359	55364			unmodified **1**	dimer
	+ enmetazobactam	55989	55992	+630	+628	imine/enamine **3, 4, or 5**	or equal mass species (**2**)
	+tazobactam	55957	55964	+598	+600	imine/enamine **3, 4, or 5**	or equal mass species (**2**)
	**TEM-116**	32102	32103			unmodified **1**	
	+ enmetazobactam	32416	32417	+313	+314	imine/enamine **3, 4, or 5**	or equal mass species (**2**)
	+ tazobactam	32404	32403	+301	+300	imine/enamine **3, 4, or 5**	or equal mass species (**2**)

[a]Masses were derived from deconvolution of measured spectra using the maximum entropy algorithm either in the *MassHunter Workstation Qualitative Analysis B.07.00 program (Agilent Technologies) for SPE-MS, or MassLynx V.4.1 (Waters) for LC-MS and native-MS.*
[b]Relative to the measured unmodified protein mass. [c]Proposed product structures are defined in Figure 2 and Figure S17A.

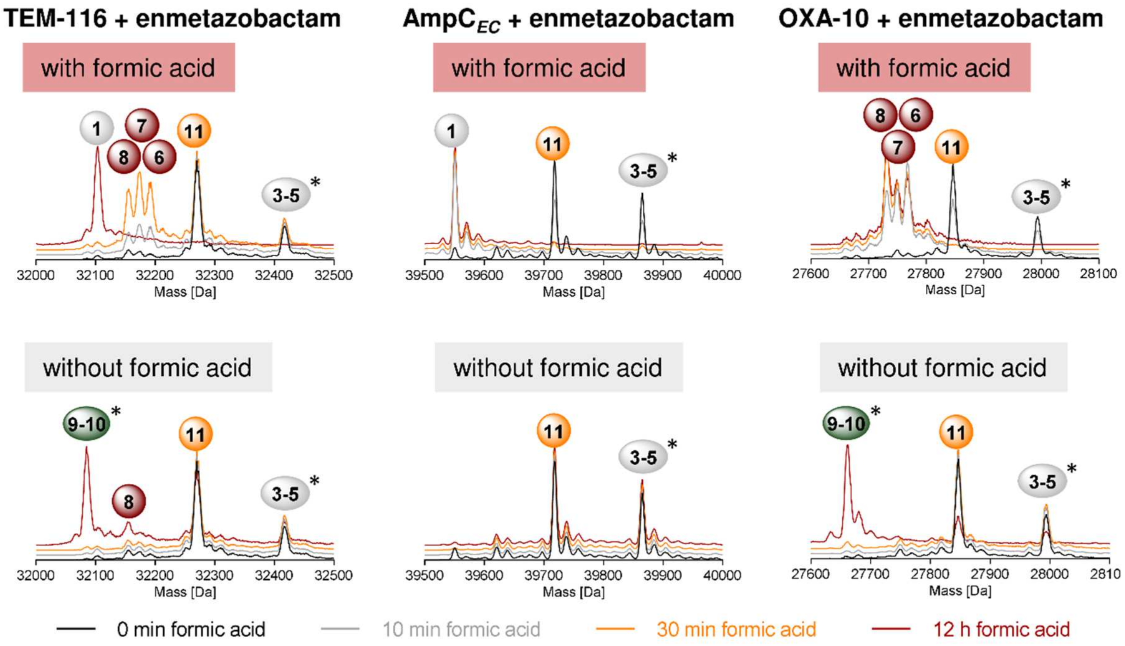

Figure S8: Effect of the addition of formic acid prior to SPE-MS analysis on modifications to TEM-116, AmpC$_{EC}$, and OXA-10 caused by reaction with enmetazobactam. TEM-116, AmpC$_{EC}$, and OXA-10 (3 µM) were reacted with a 100-fold excess of enmetazobactam in 50 mM Tris pH 7.5. Further reaction was induced by addition of formic acid to a final concentration of 0.1 % (v/v). Samples were analyzed using SPE-MS. Deconvoluted spectra, obtained using the maximum entropy algorithm in the MassHunter Workstation Qualitative Analysis B.07.00 program (Agilent), are shown. Proposed structures corresponding to the labelled mass shifts are given in Figure 2, Figure S17A, and Table S5. *Note the mass shifts may reflect more than one species, e.g. **2-5** as well as **9**-**10** give the same mass shift. On addition of formic acid, fragmentation of the enmetazobactam-derived TEM-116- and OXA-10-complexes was observed (top). Evidence for such reaction was not observed without formic acid addition (bottom) over the same time-frame. Note that faster deacylation was observed for the AmpC$_{EC}$-complexes with formic acid treatment (compared to its absence). The slower / less extensive fragmentation induced by adding formic acid to the inhibitor complex prior to SPE-MS analysis compared to that observed during LC-MS analysis, may result from partial buffering under the assay conditions compared to the acetonitrile/water mixture used as a mobile phase in the LC.

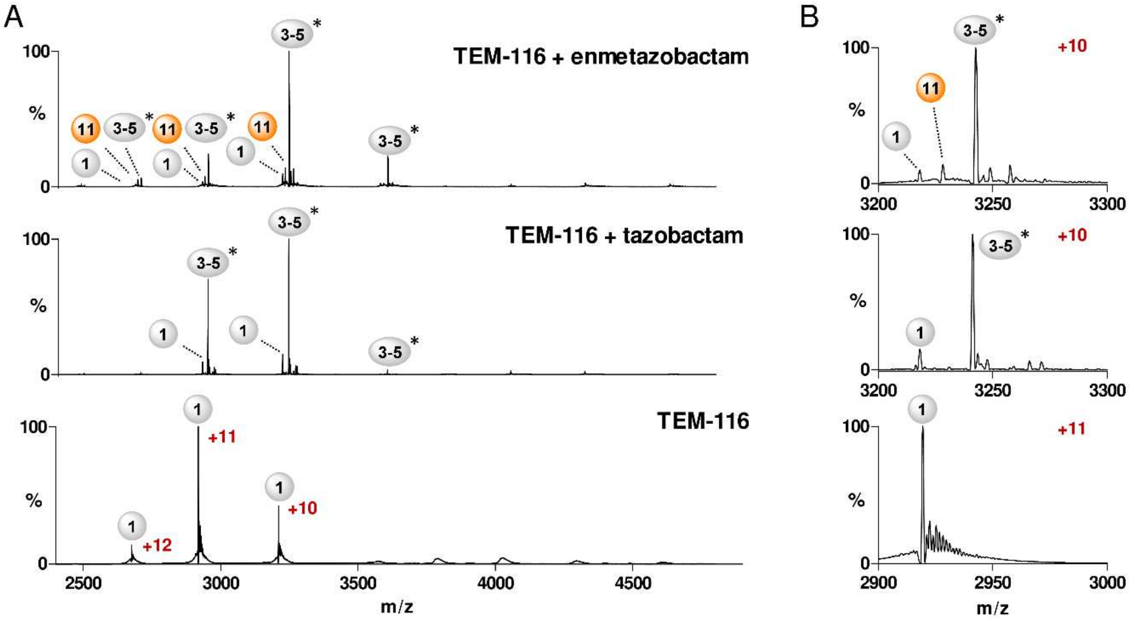

Figure S9: Native-MS studies on the interaction of TEM-116 with tazobactam and enmetazobactam after 20 min incubation. TEM-116 (10 µM) was incubated with tazobactam or enmetazobactam (1 mM) in 1 M ammonium acetate, pH 7.5 at r.t. for 20 min. Samples were desalted (using a Zeba micro spin desalting column, 7K MWCO, Thermo Scientific) immediately prior to analysis and sprayed directly into the mass-spectrometer. (A) Mass spectra of unmodified TEM-116, with tazobactam, and with enmetazobactam. Charge states are in red. Note: The lower charge state observed on binding of tazobactam and enmetazobactam (major charge state: +10) compared to unmodified TEM-116 (major charge state: +11) indicates binding of the inhibitors leads to closer conformation. (B) Magnification of the major charge states of the respective spectra in (A). Proposed structures corresponding to the labelled mass shifts are given in Figure 2, Figure S17A, and Table S5. *Note mass shifts may reflect more than one species, e.g. **2**-**5** give the same mass shift.

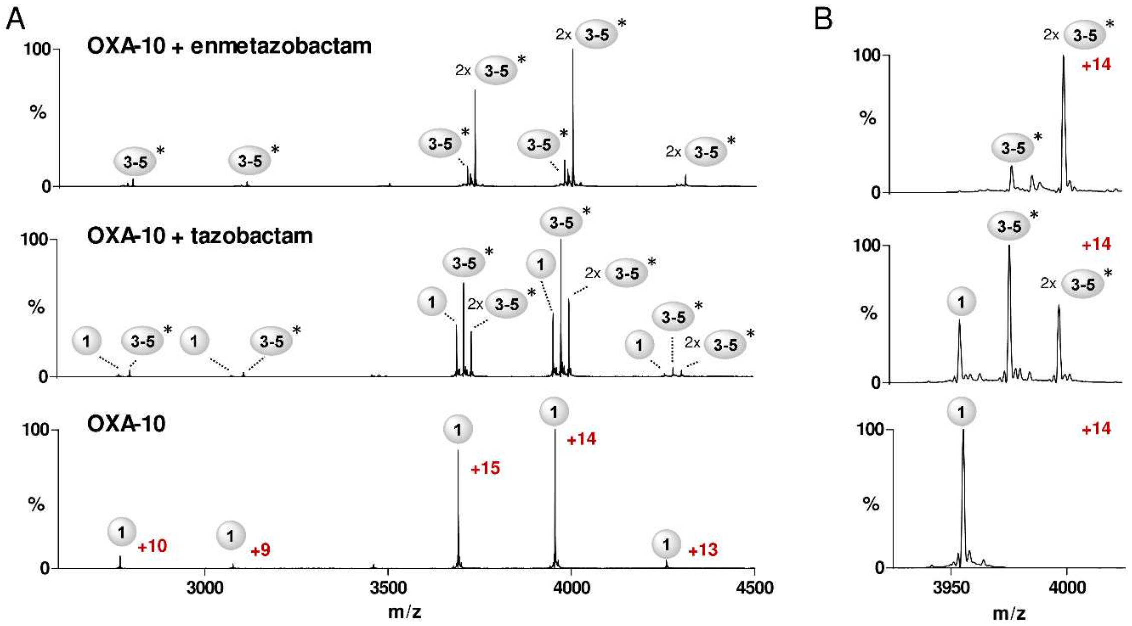

Figure S10: Native-MS studies on the interaction of OXA-10 with tazobactam and enmetazobactam after 20 min incubation. OXA-10 (10 µM) was incubated with tazobactam or enmetazobactam (1 mM) in 1 M ammonium acetate, pH 7.5 at r.t. for 20 min. Samples were desalted (using a Zeba micro spin desalting column, 7K MWCO, Thermo Scientific) immediately prior to analysis and sprayed directly into the mass-spectrometer. (A) Mass spectra of unmodified OXA-10, with tazobactam, and with enmetazobactam. Charge states are in red. Note: In agreement with reported studies(3) OXA-10 was observed in a predominantly dimeric form. (B) Magnification of the major charge state (+ 14) of the respective spectra in (A). Proposed structures corresponding to the labelled mass shifts are given in Figure 2, Figure S17A, and Table S5. *Note mass shifts may reflect more than one species, e.g. **2-5** give the same mass shift.

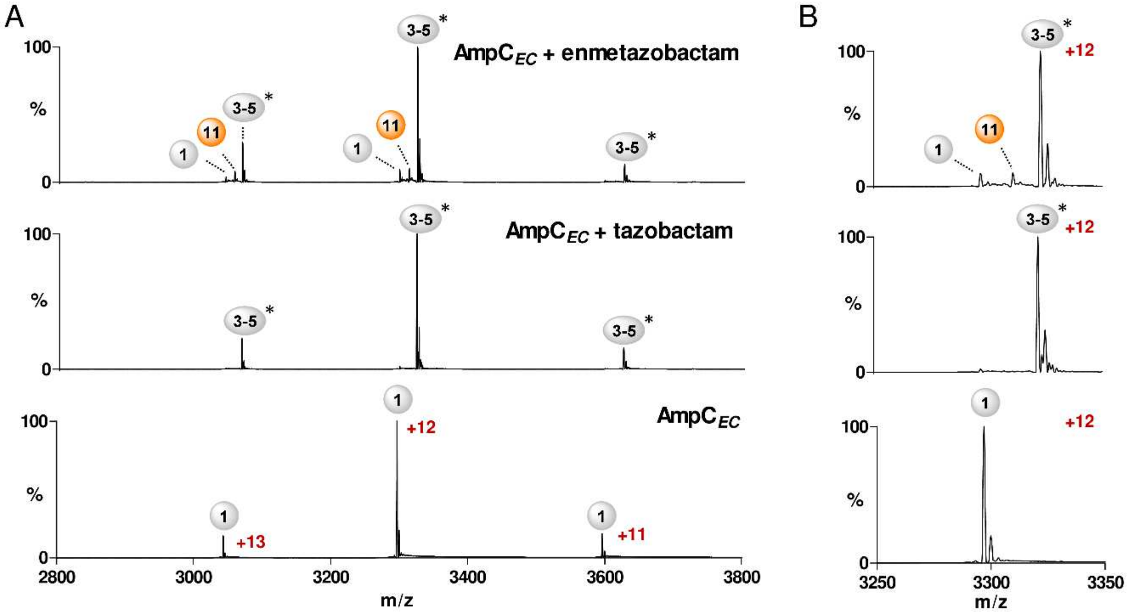

Figure S11: Native-MS studies on the interaction of AmpC$_{EC}$ with tazobactam and enmetazobactam after 20 min incubation. AmpC$_{EC}$ (10 µM) was incubated with tazobactam or enmetazobactam (1 mM) in 1 M ammonium acetate, pH 7.5 at r.t. for 20 min. Samples were desalted (using a Zeba micro spin desalting column, 7K MWCO, Thermo Scientific), immediately prior to analysis and sprayed directly into the mass-spectrometer. (A) Mass spectra of unmodified AmpC$_{EC}$, with tazobactam, and with enmetazobactam. Charge states are in red. (B) Magnification of the major charge state (+ 12) of the respective spectra in (A). Proposed structures corresponding to the labelled mass shifts are given in Figure 2, Figure S17A, and Table S5. *Note mass shifts may reflect more than one species, e.g. **2-5**, give the same mass shift.

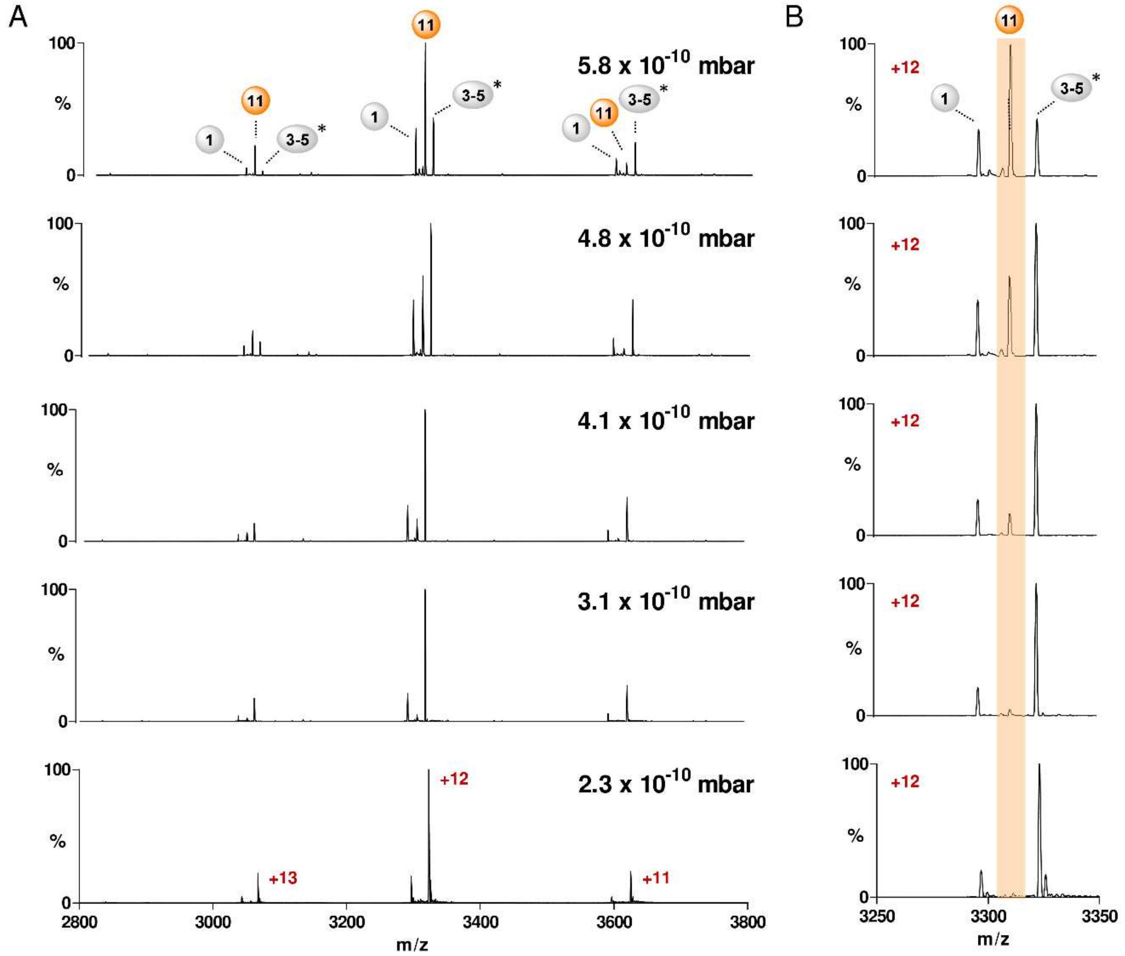

Figure S12: Native-MS studies showing the dependence of elimination from the AmpC*EC*-enmetazobactam derived complex on gas-pressure. AmpC*EC* (10 µM) was incubated with enmetazobactam (1 mM) in 1 M ammonium acetate, pH 7.5 at r.t. for 20 min. The sample was desalted (using a Zeba micro spin desalting column, 7K MWCO, Thermo Scientific) immediately prior to analysis and sprayed directly into the mass-spectrometer. Spectra were acquired without collisional activation and at the indicated UHV gas pressures. (A) Mass spectra of the AmpC*EC*-enmetazobactam derived complex with varying UHV gas pressures. Charge states are in red. (B) Magnification of the major charge state (+ 12) of the respective spectra in (A). Proposed structures corresponding to the labelled mass shifts are given in Figure 2, Figure S17A, and Table S5. *Note mass shifts may reflect more than one species, e.g. **2-5** give the same mass shift.

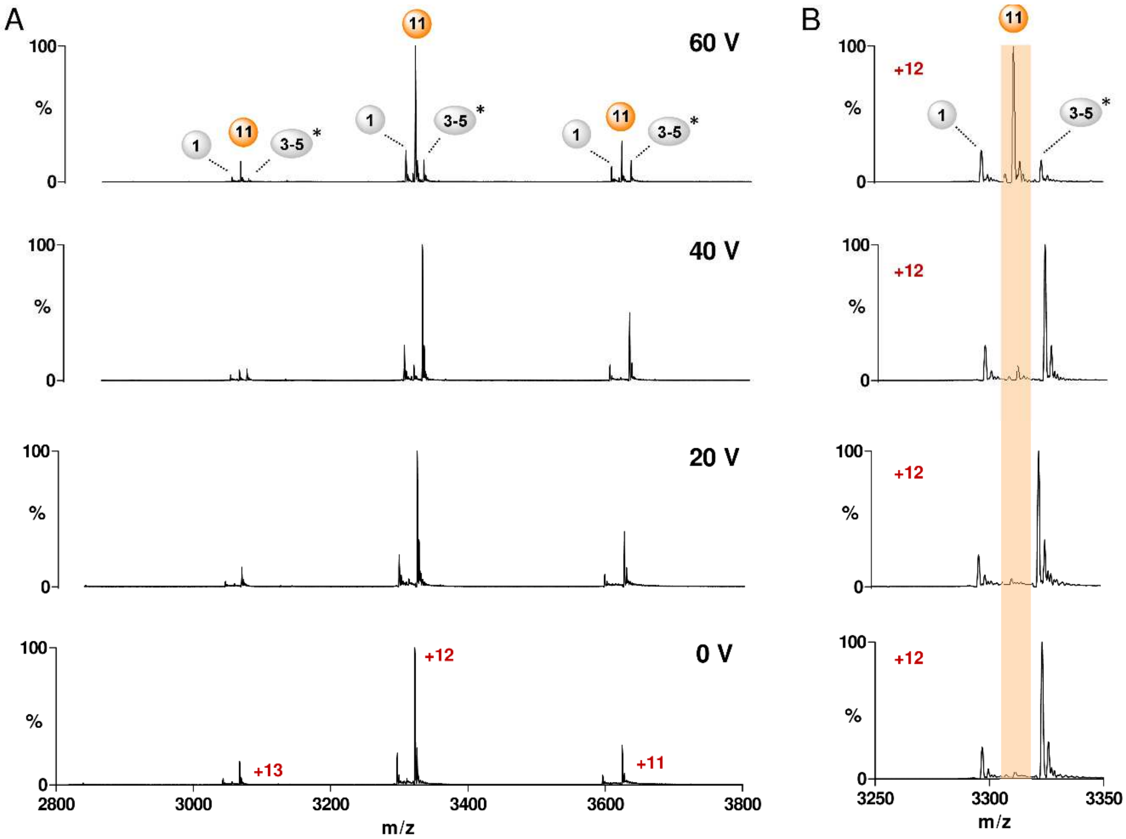

Figure S13: Native-MS studies showing the effect of collisional activation in the higher-energy C-trap dissociation (HCD) cell on formation of elimination product 11 from the AmpC*EC*-enmetazobactam derived complex. AmpC*EC* (10 µM) was incubated with enmetazobactam (1 mM) in 1 M ammonium acetate, pH 7.5 at r.t. for 20 min. The sample was desalted (using a Zeba micro spin desalting column, 7K MWCO, Thermo Scientific) immediately prior to analysis and sprayed directly into the spectrometer. Spectra were acquired at a constant UHV gas-pressure of 2.7 x 10^{-10} mbar and at the indicated HCD collisional activation energy. (A) Mass spectra of the AmpC*EC*-enmetazobactam derived complex at a constant gas pressure and varying collisional activation energy. Charge states are in red. (B) Magnification of the major charge state (+ 12) of the respective spectra in (A). Proposed structures corresponding to the labelled mass shifts are given in Figure 2, Figure S17A, and Table S5. *Note mass shifts may reflect more than one species, e.g. **2-5** give the same mass shift.

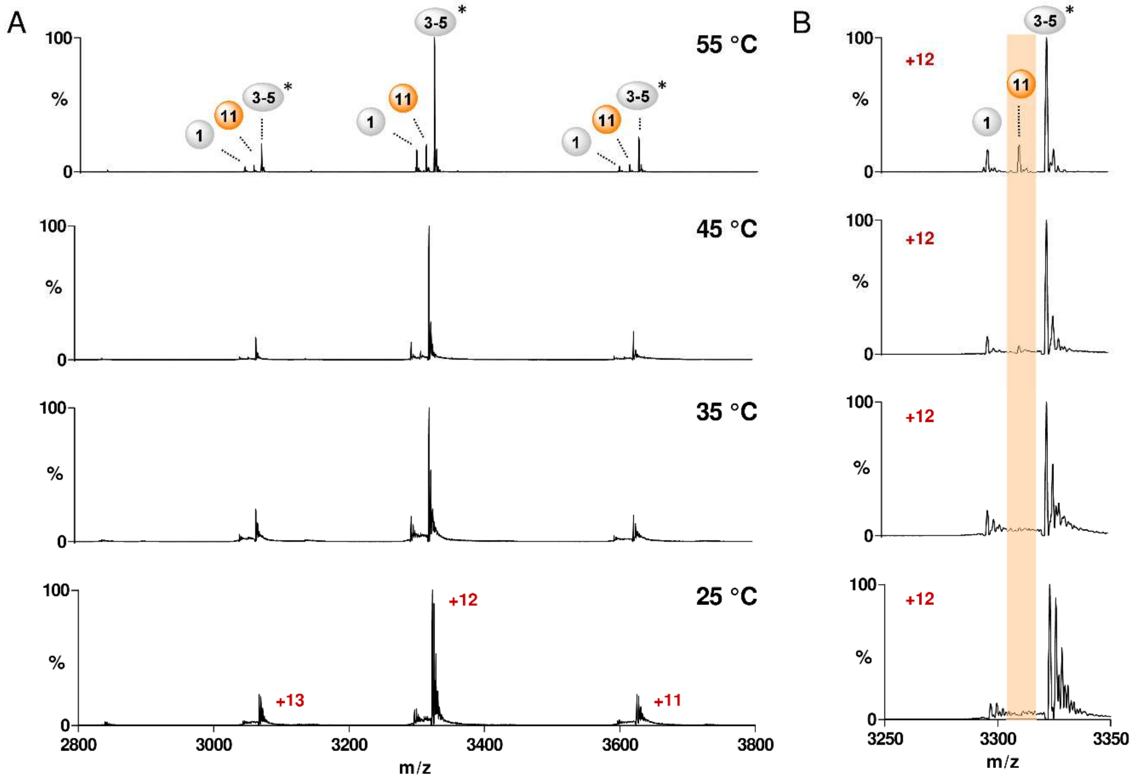

Figure S14: Native-MS studies showing the dependence of elimination from the AmpC$_{EC}$-enmetazobactam derived complex on solution temperature. AmpC$_{EC}$ (10 µM) was incubated with enmetazobactam (1 mM) in 1 M ammonium acetate, pH 7.5 at r.t. for 20 min, then for 1 h at the indicated temperatures. Samples were desalted (using a Zeba micro spin desalting column, 7K MWCO, Thermo Scientific) immediately prior to analysis and sprayed directly into the mass-spectrometer. Spectra were acquired without collisional activation at a constant UHV gas-pressure of 2.7 x 10^{-10} mbar. (A) Mass spectra of AmpC$_{EC}$-enmetazobactam derived complex incubated at the indicated temperatures. Charge states are iin red. (B) Magnification of the major charge state (+ 12) of the respective spectra in (A). Proposed structures corresponding to the labelled mass shifts are given in Figure 2, Figure S17A, and Table S5. *Note mass shifts may reflect more than one species, e.g. **2-5** give the same mass shift.

Table S6: Data collection and refinement statistics for the AmpC$_{EC}$ – enmetazobactam derived crystal.

**Datasets**	**AmpC$_{EC}$ –enmetazobactam** (PDB: 6T35)
**Data Collection**	
Beamline (Wavelength, Å)	DLS I03 (0.9763)
Detector	Pilatus 6M-F
Data Processing	Xia2
Space group	$P\,4_3\,3\,2$
Cell dimensions	
a,b,c (Å)	138.0, 138.0, 138.0
α, β, γ (°)	90, 90, 90
No. of molecules/ASU	1
No. reflections	45784 (3313)*
Resolution (Å)	56.34-1.75 (1.78-1.75)*
R_{merge}(I)	0.128 (5.437)*
I/σI	28.1 (1.3)*
$CC_{1/2}$	1.00 (0.53)*
Completeness (%)	100 (100)*
Multiplicity	77.5 (81.1)*
Wilson B value (Å^2)	31.19
**Refinement**	PHENIX
R_{work}/R_{free}	0.1667/0.1844
No. atoms	
- Enzyme	2724
- Ser64-enmetazobactam	28
- Water	258
Average B-factors	
- Enzyme	38.17
- Ser64-enmetazobactam	59.29
- Water	45.64
RMS$^\$$ deviations	
- Bond lengths (Å)	0.005
- Bond angles (°)	0.80

$^\#$ ASU = asymmetric unit.

$^\$$ RMS = root mean square.

*Highest resolution shell in parentheses.

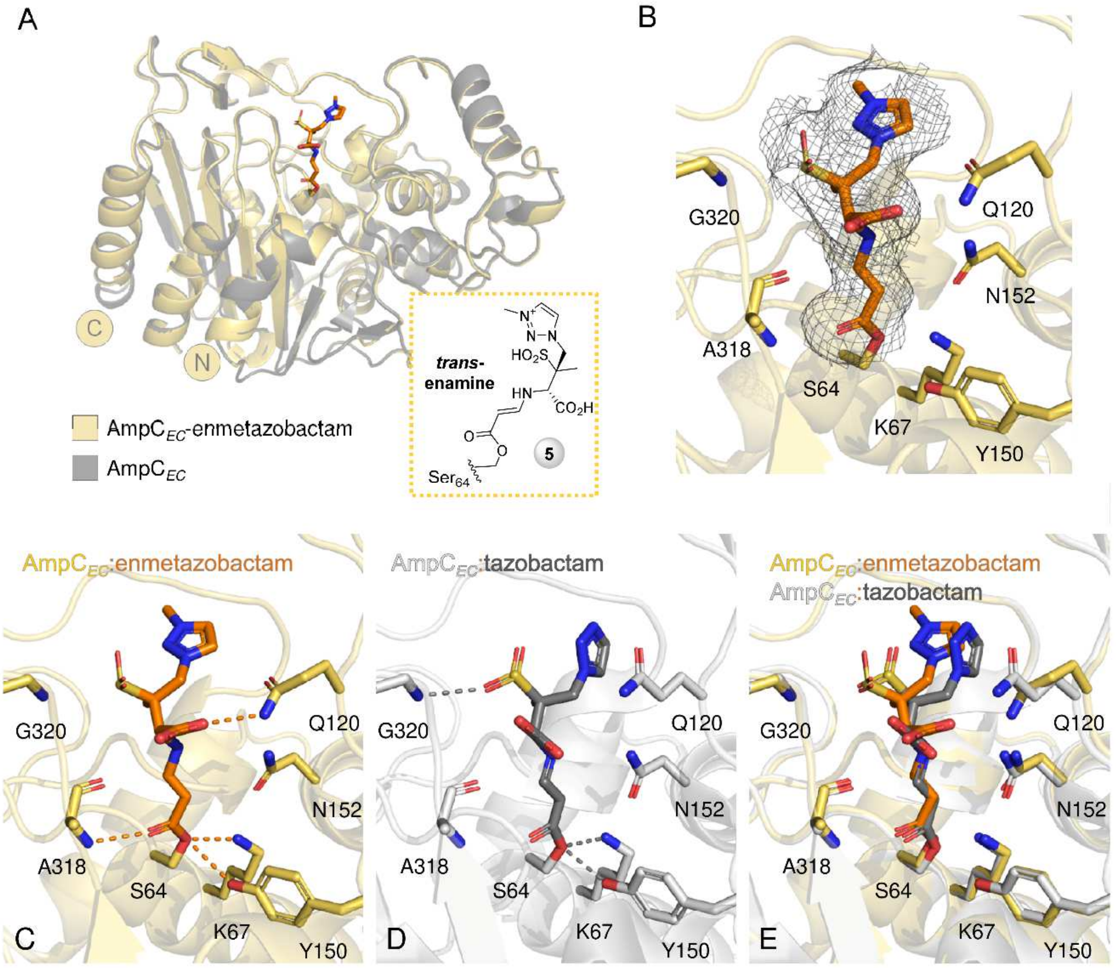

Figure S15: Views from crystal structures of AmpC$_{EC}$ reacted with enmetazobactam (PDB: 6T35) and tazobactam (PDB 6WA7 (4)) to give (predominantly) a *trans*-enamine linked to Ser64. (A) Comparison of overviews of the unmodified AmpC$_{EC}$ fold (PDB: 6T3D (5), grey) and AmpC$_{EC}$ in complex with the enmetazobactam derived *trans*-enamine (yellow, **5** in Figure 2 and Figure S17A) shows no substantial differences in the overall fold (backbone RMSD: 0.18 Å). (B) mF$_o$-DF$_c$ polder OMIT maps(6) contoured at 3.0 σ and carved around the enmetazobactam derived *trans*-enamine bound to Ser64. (C) Interaction of AmpC$_{EC}$ with the enmetazobactam derived sulfinic acid *trans*-enamine (orange, PDB: 6T35). (D) Interaction of AmpC$_{EC}$ with the tazobactam derived sulfinic acid *trans*-enamine (dark gray, PDB 6WA7 (4)). Hydrogen bonding interactions are indicated as dashes. (E) Superimposition of the two *trans*-enamine complexes (color coding as in (C) and (D)) with uncomplexed AmpC$_{EC}$ (PDB: 6T3D (5)). The β-lactam derived carbonyl of enmetazobactam is positioned to interact with the backbone NH of Ala318, (2.9 Å); one of the enmetazobactam derived C-2 carboxylate oxygens is proximate to the sidechain amide nitrogen of Gln120 (2.5 Å). The triazole *N*-methyl group does not appear to interact directly with any active site residues; it is rotated by ~90 ° compared to the triazole motif as observed in a recently reported structure of an AmpC$_{EC}$-tazobactam derived *trans*-enamine complex (4).

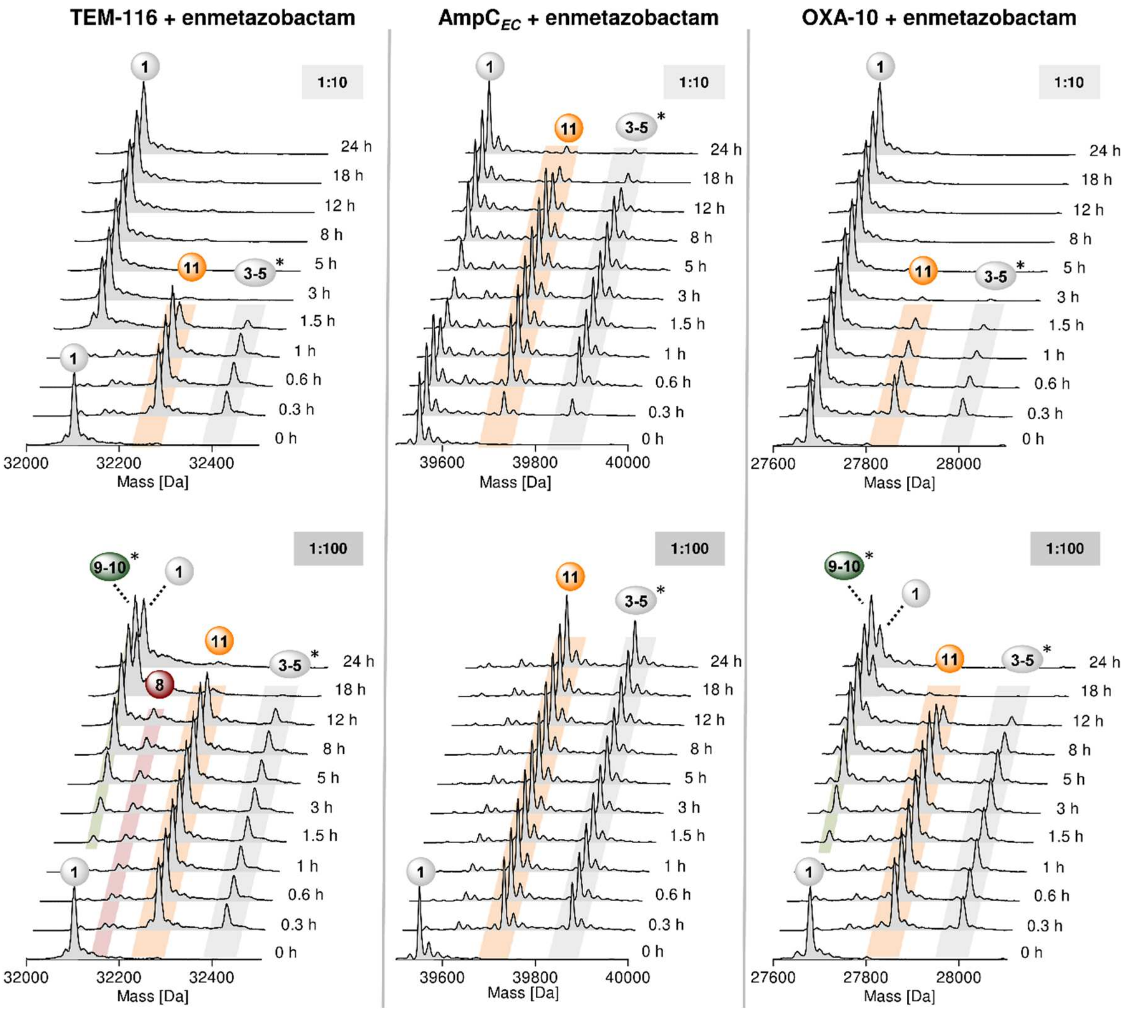

Figure S16: SPE-MS time courses of TEM-116, AmpC$_{EC}$, and OXA-10 modification by enmetazobactam. TEM-116, AmpC$_{EC}$, and OXA-10 (3 µM) were incubated with a 10 or 100 molar excess of enmetazobactam at r.t. Buffer: 50 mM Tris buffer pH 7.5. Spectra were acquired without the inhibitor (0 h) and after incubation for the indicated times. Deconvoluted spectra, obtained using the maximum entropy algorithm in the MassHunter Workstation Qualitative Analysis B.07.00 program (Agilent), are shown. Proposed structures corresponding to the labelled mass shifts are given in Figure 2, Figure S17A, and Table S5. *Note mass shifts may reflect more than one species, e.g. **2**-**5** as well as **9**-**10** give the same mass shift.

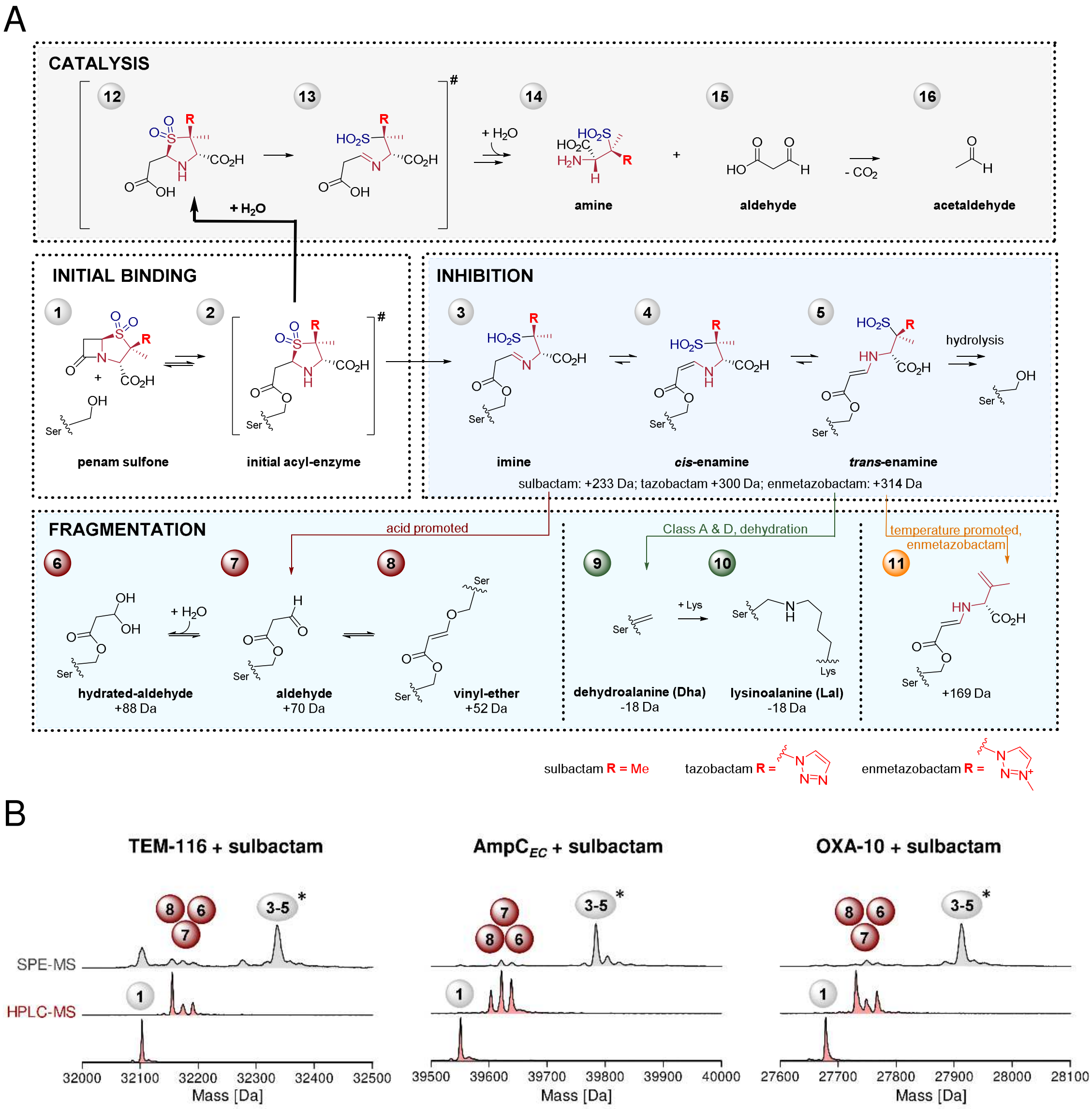

Figure S17: Results from protein-observed MS analysis of reactions of SBLs with sulbactam. (A) Proposed product structures for the reaction of sulbactam with TEM-116, AmpC*EC*, and OXA-10. Note: This is the same scheme as in Figure 2. (B) Protein-observed MS analysis. For LC-MS analyses, TEM-116, AmpC*EC*, and OXA-10 (1 µM) were incubated with sulbactam (500 µM) and for SPE-MS analyses, TEM-116, AmpC*EC*, and OXA-10 (3 µM) were incubated with sulbactam (300 µM) in 50 mM tris pH 7.5 at r.t. for 20 min. In the LC-method modified proteins were eluted using a mobile phase of 0.1 % (v/v) aqueous formic acid over approx. 4-5 min; elution using the SPE-method was done using the same mobile phase within 4 sec. See Table S5 for a summary of the calculated and observed protein masses and mass shifts. *Note mass shifts may reflect more than one species, e.g. **2**-**5** give the same mass shift.

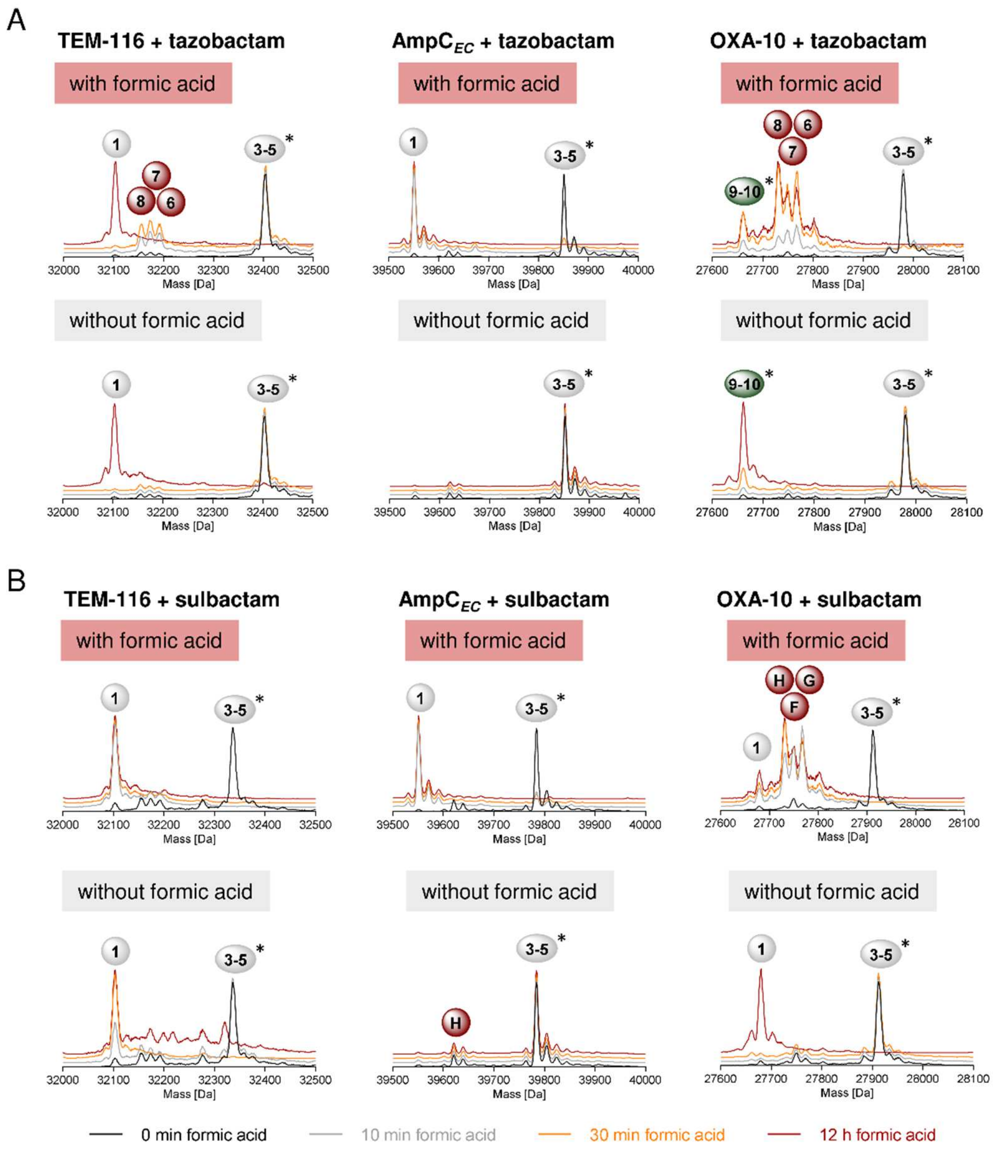

Figure S18: Effect of the addition of formic acid addition prior to SPE-MS analysis on modifications of TEM-116, AmpC*EC* and OXA-10 caused by reaction with tazobactam or sulbactam. TEM-116, AmpC*EC*, and OXA-10 (3 μM) were reacted with a 100-fold molar excess of tazobactam or a 500-fold molar excess of sulbactam in 50 mM Tris pH 7.5. Further reaction was induced by addition of formic acid to a final concentration of 0.1 % (v/v). Samples were analyzed using the SPE-MS method. Deconvoluted spectra, obtained using the maximum entropy algorithm in the MassHunter Workstation Qualitative Analysis B.07.00 program (Agilent), are shown. Proposed structures corresponding to the labelled mass shifts are given in Figure 2, Figure S17A, and Table S5. *Note mass shifts may reflect more than one species, e.g. **2-5** as well as **9**-**10** give the same mass shift. On addition of formic acid, fragmentation of the tazobactam- and sulbactam-derived OXA-10 and TEM-116 complexes was observed (top). Such reaction was not observed without formic acid addition (bottom) over the same time-frame. Note that faster deacylation was observed for all other sulbactam-derived complexes with formic acid treatment (compared to its absence). The slower / less extensive fragmentation induced by adding formic acid to the inhibitor complex prior to SPE-MS analysis compared to that observed during LC-MS analysis, may result from partial buffering under the assay conditions compared to the acetonitrile/water mixture used as mobile phase in the LC assays.

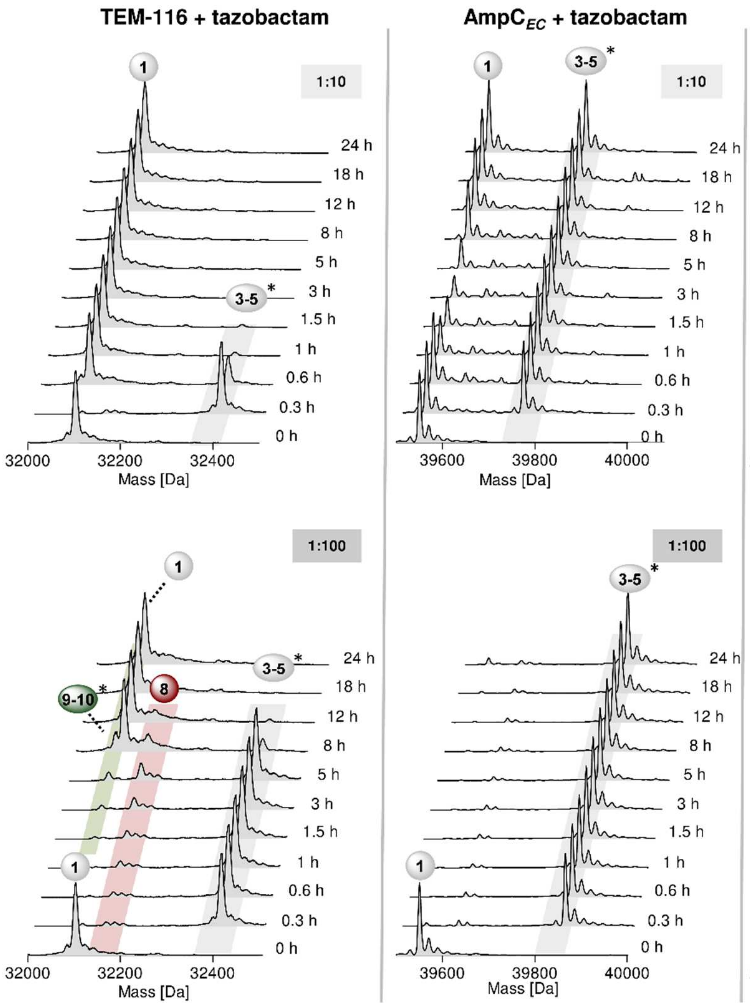

Figure S19: SPE-MS time courses of TEM-116, AmpC*EC*, and OXA-10 modification by tazobactam. TEM-116, AmpC*EC*, and OXA-10 (3 µM) were incubated with a 10- or 100-fold excess of tazobactam at r.t. Buffer: 50 mM Tris buffer pH 7.5. Mass spectra were acquired without the inhibitor (0 h) and after incubation for the indicated times. Deconvoluted spectra, obtained using the maximum entropy algorithm in the MassHunter Workstation Qualitative Analysis B.07.00 program (Agilent), are shown. Proposed structures corresponding to the labelled mass shifts are given in Figure 2, Figure S17A, and Table S5. *Note mass shifts may reflect more than one species, e.g. **2-5** as well as **9**-**10** give the same mass shift.

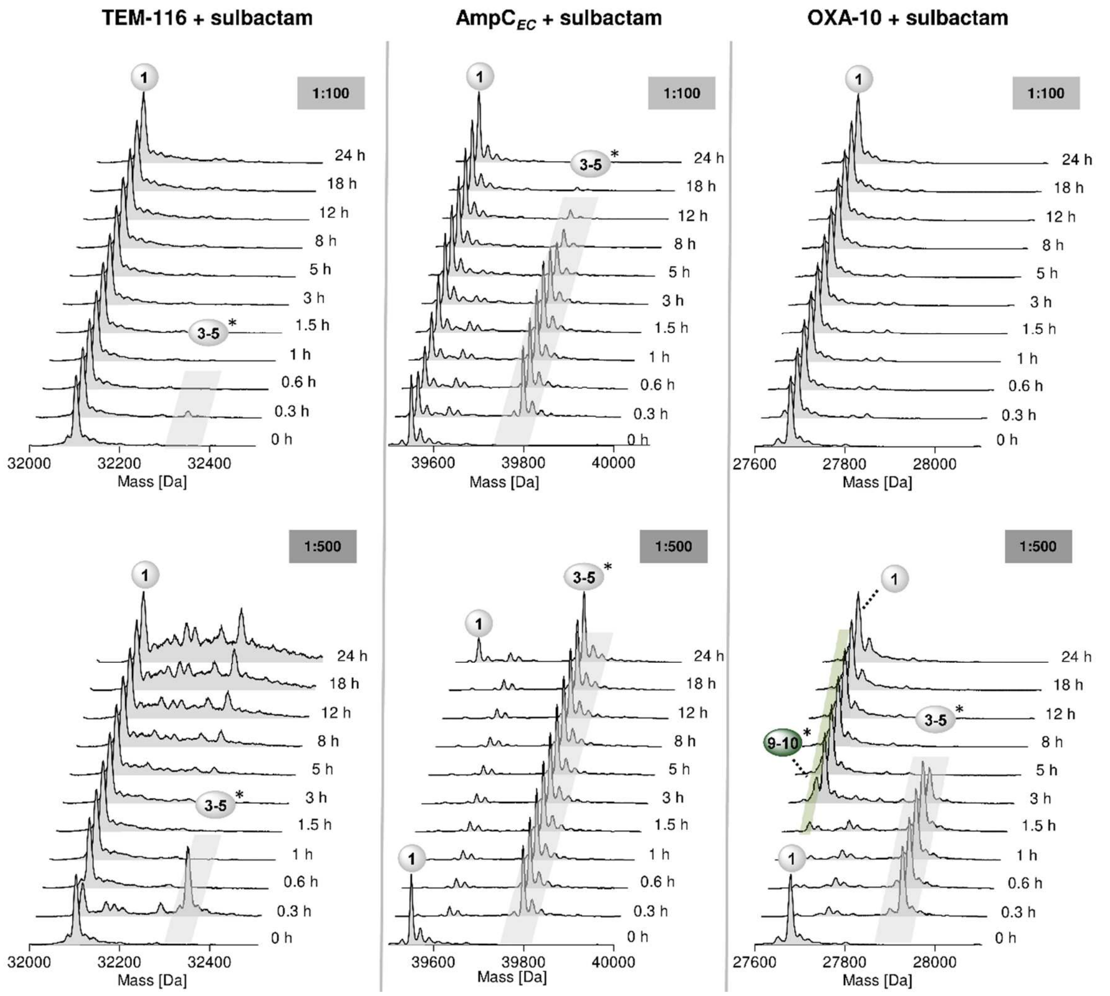

Figure S20: SPE-MS time courses of TEM-116, AmpC$_{EC}$, and OXA-10 modification by sulbactam. TEM-116, AmpC$_{EC}$, and OXA-10 (3 µM) were incubated with a 100- or 500-fold excess of sulbactam at r.t. Buffer: 50 mM Tris buffer pH 7.5. Mass spectra were acquired without the inhibitor (0 h) and after incubation for the indicated times. Deconvoluted spectra, obtained using the maximum entropy algorithm in the MassHunter Workstation Qualitative Analysis B.07.00 program (Agilent), are shown. Proposed structures corresponding to the labelled mass shifts are given in Figure 2, Figure S17A, and Table S5. *Note mass shifts may reflect more than one species, e.g. **2-5** as well as **9**-**10** give the same mass shift.

A

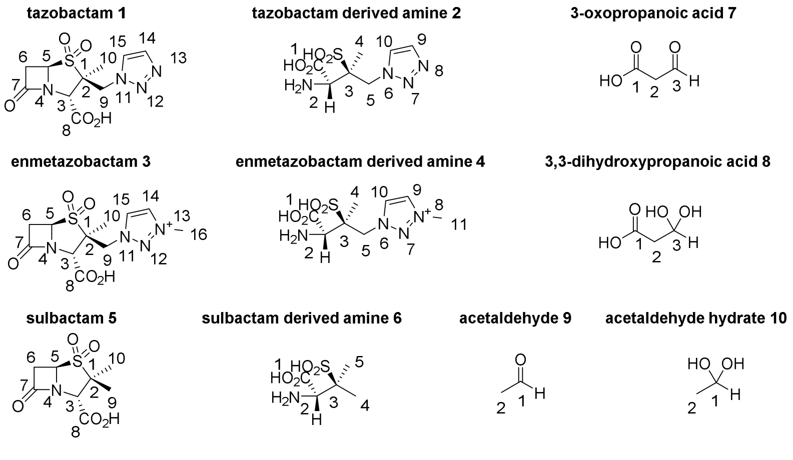

B

tazobactam 1

Position	^1H (ppm)
1	-
2	-
3	4.37 (s)
4	-
5	4.93 (d, J = 4.3 Hz)
6	3.59 (dd, J = 17.2, 4.3 Hz), 3.37 (dd, J= 17.4, 1.4 Hz)
7	-
8	-
9	5.24 (d, J = 16.0 Hz), 5.06 (d, J = 16.0 Hz)
10	1.31 (s)
11	-
12	-
13	-
14	7.76 (d, J = 1.1 Hz)
15	8.04 (d, J = 1.1 Hz)

enmetazobactam 3

Position	^1H (ppm)
1	-
2	-
3	4.44 (s)
4	-
5	5.00 (s)
6	3.63 (dd, J = 18.3, 3.5 Hz), 3.39 (d, J = 18.3 Hz)
7	-
8	-
9	5.40 (d, J = 15.4 Hz), 5.27 (d, J = 15.6 Hz)
10	1.43 (s)
11	-
12	-
13	-
14	8.66 (s)
15	8.55 (s)
16	4.31 (s)

sulbactam 5

Position	^1H (ppm)
1	-
2	-
3	4.1 (s)
4	-
5	4.90 (s)
6	3.55 (dd, J = 18.0, 3.8 Hz), 3.30 (d, J = 18.1 Hz)
7	-
8	-
9	1.35 (s)
10	1.49 (s)

3-oxopropanoic acid 7 (reported(7))

Position	^1H (ppm)
1	-
2	3.31 (d, J = 2.9 Hz)
3	9.61 (t, J = 2.9 Hz)

tazobactam derived amine 2

Position	^1H (ppm)
1	-
2	4.11 (s)
3	-
4	1.09 (s)
5	-
6	-
7	-
8	-
9	7.71 (d, J = 1.1 Hz)
10	7.98 (d, J = 1.1 Hz)

enmetazobactam derived amine 4

Position	^1H (ppm)
1	-
2	4.04 (s)
3	-
4	1.12 (s)
5	-
6	-
7	-
8	-
9	8.61 (s)
10	8.43 (s)
11	4.27 (s)

sulbactam derived amine 6

Position	^1H (ppm)
1	-
2	3.91 (s)
3	-
4	1.00 (s)
5	1.07 (s)

3,3-dihydroxypropanoic acid 8 (reported(7))

Position	^1H (ppm)
1	-
2	2.41 (d, J = 5.6 Hz)
3	5.24 (t, J = 5.6 Hz)

acetaldehyde 9 (reported(7))

Position	^1H (ppm)
1	9.58 (q, J = 3.0 Hz)
2	2.14, (d, J = 3.1 Hz)

acetaldehyde hydrate 10 (reported(7))

Position	^1H (ppm)
1	5.16 (q, J = 5.3 Hz)
2	1.23 (d, J = 5.3 Hz)

Figure S21: Chemical shift assignments for tazobactam, enmetazobactam, sulbactam and their products. (A) Structures of inhibitors and assigned products. (B) Chemical shift assignments. Spectra (750 MHz) were recorded in 50 mM phosphate buffer, pH 7.5, 10 % D_2O. Chemical shifts and assignments for 3-oxopropanoic acid 7, 3,3-dihydroxypropanoic acid 8, acetaldehyde 9 and acetaldehyde hydrate 10 are in agreement with reported data (7).

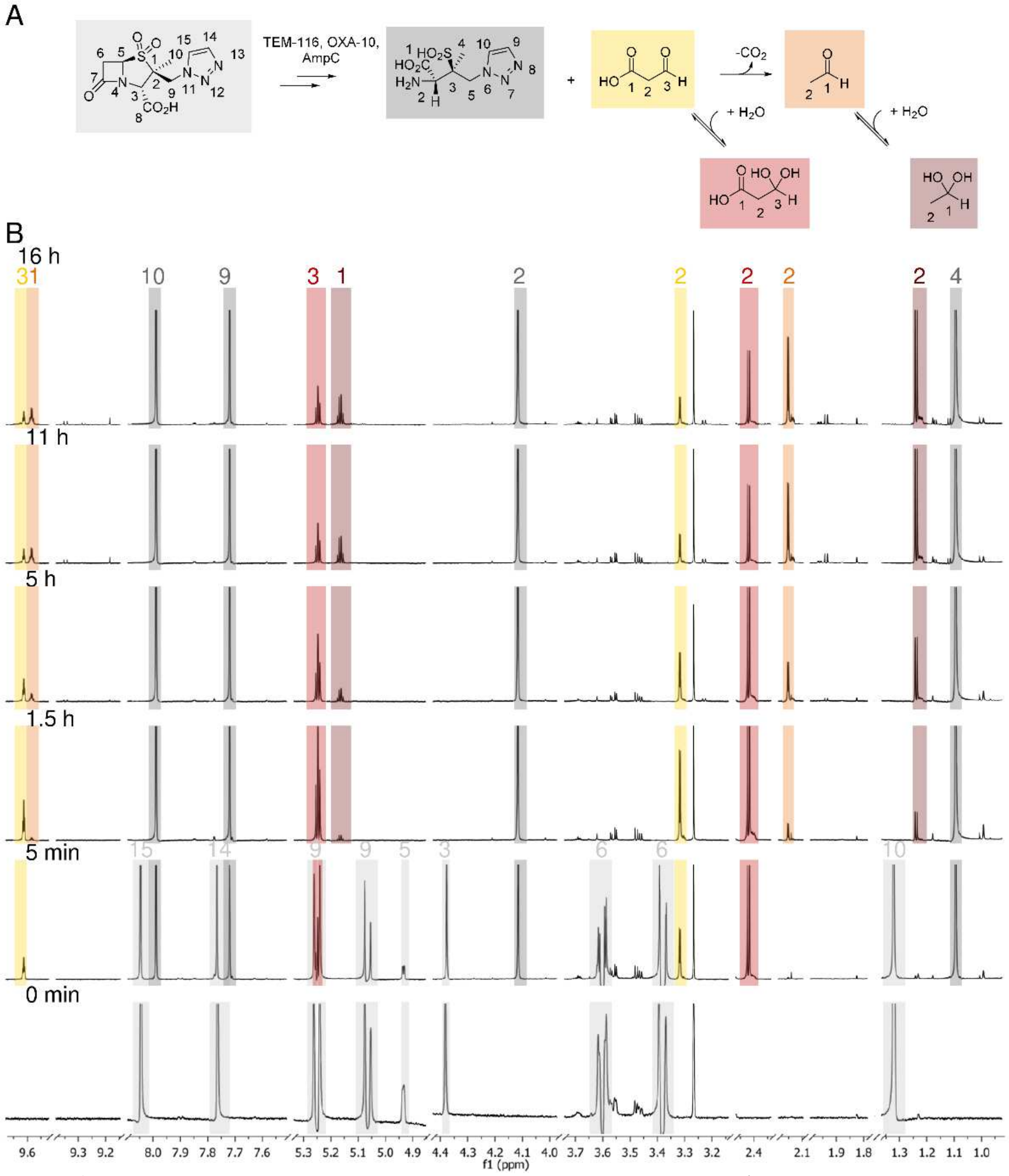

Figure S22: ^{1}H NMR analysis of the reaction of tazobactam with OXA-10. ^{1}H NMR Spectra (750 MHz) for the products of tazobactam formed by reaction with OXA-10 and likely subsequent non-enzymatic reactions. OXA-10 (10 µM) was incubated with tazobactam (5 mM, r.t., 50 mM phosphate buffer pH 7.5, 10 % D_2O). Chemical shift assignments are shown in Figure S21.

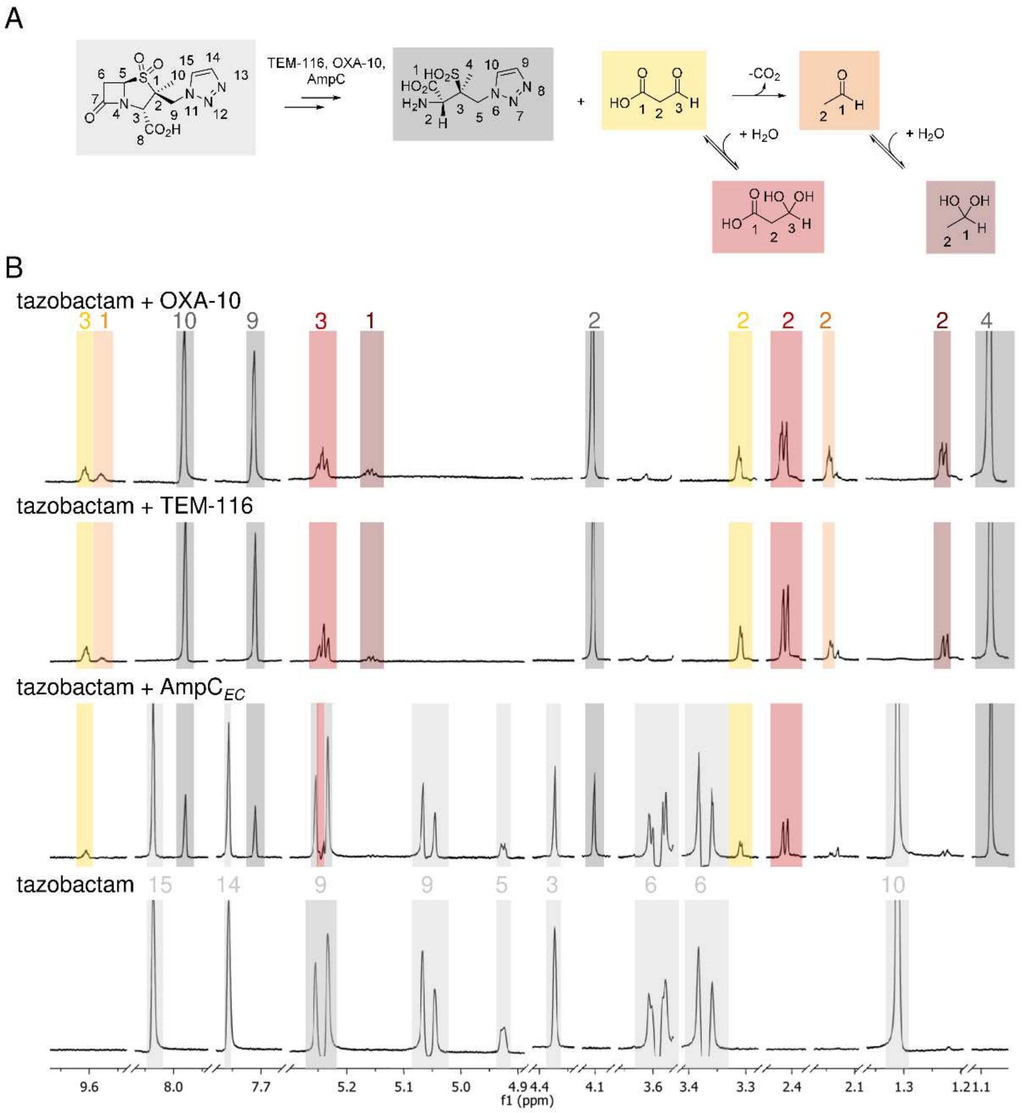

Figure S23: ^{1}H NMR analysis of the reaction of tazobactam with OXA-10, TEM-116, or AmpC$_{EC}$. ^{1}H NMR spectra (750 MHz) for the products of tazobactam formed by reaction with SBLs and likely subsequent non-enzymatic reactions. OXA-10, TEM-116, or AmpC$_{EC}$ (10 μM) were incubated with tazobactam (1 mM, 18 h, r.t., 50 mM phosphate buffer pH 7.5, 10 % D_2O). In all cases the inhibitors gave the same major reaction products. Chemical shift assignments are shown in Figure S21.

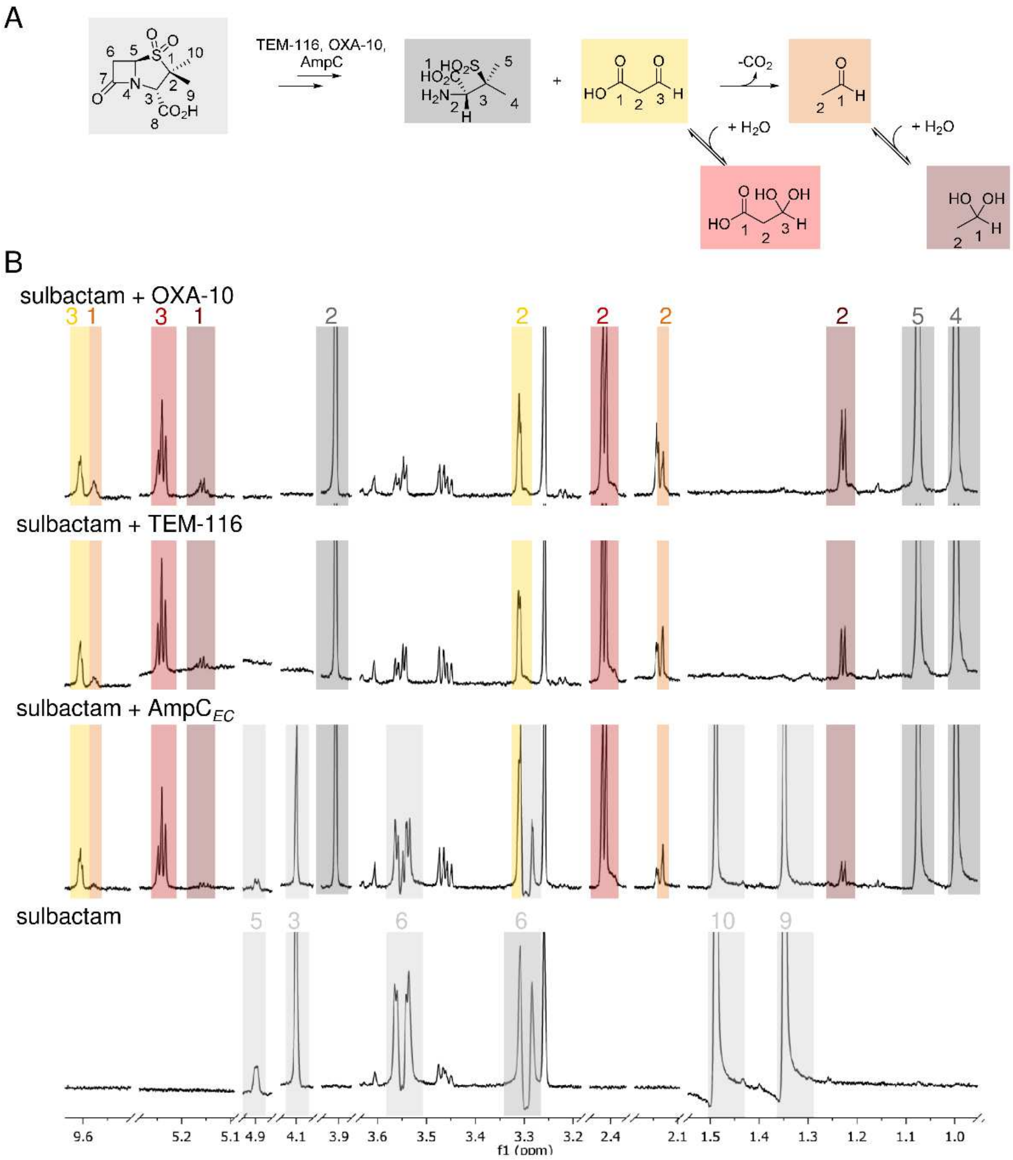

Figure S24: ^{1}H NMR analysis of the reaction of sulbactam with OXA-10, TEM-116, or AmpC$_{EC}$. ^{1}H NMR spectra (750 MHz) for the products of sulbactam formed by reaction with SBLs and likely subsequent non-enzymatic reactions. OXA-10, TEM-116, or AmpC$_{EC}$ (10 µM) were incubated with sulbactam (1 mM, 18 h, r.t., 50 mM phosphate buffer pH 7.5, 10 % D_2O). In all cases the inhibitors gave the same major products. Chemical shift assignments are shown in Figure S21.

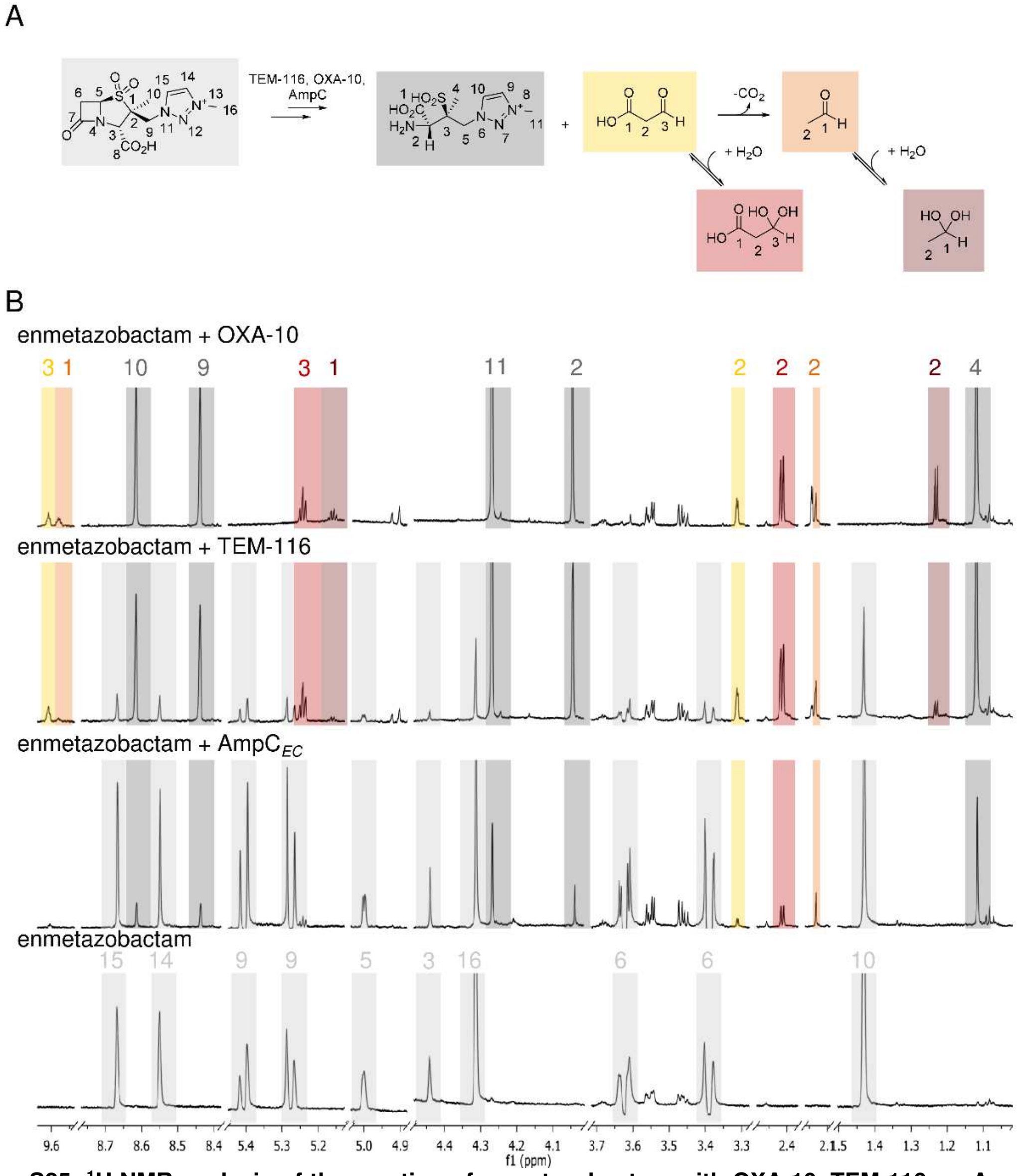

Figure S25: ^{1}H NMR analysis of the reaction of enmetazobactam with OXA-10, TEM-116, or AmpC$_{EC}$. ^{1}H NMR spectra (750 MHz) for the products of enmetazobactam formed by reaction with SBLs and likely subsequent non-enzymatic reactions. OXA-10, TEM-116, or AmpC$_{EC}$ (10 µM) were incubated with enmetazobactam (1 mM, 18 h, r.t., 50 mM phosphate buffer pH 7.5, 10 % D_2O). In all cases the inhibitors gave the same major products. Chemical shift assignments are shown in Figure S21.

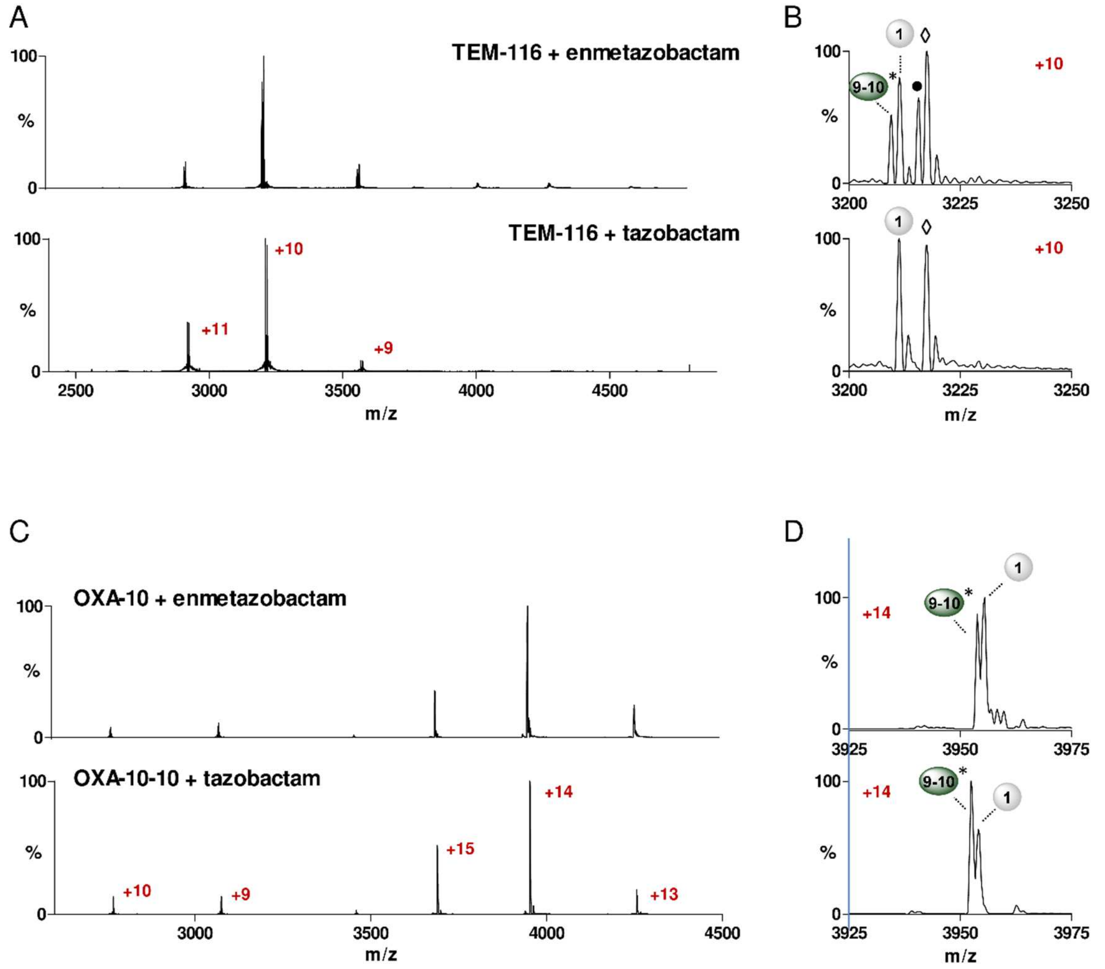

Figure S26: Non-denaturing (native) MS studies on TEM-116 or OXA-10 interactions with tazobactam and enmetazobactam after prolonged incubation. TEM-116 or OXA-10 (10 µM) were incubated with tazobactam or enmetazobactam (1 mM) in 50 mM Tris pH 7.5 at r.t. for 24 h. Prior to analysis samples were buffer exchanged into 1 M ammonium acetate, pH 7.5 ((using a Zeba micro spin desalting column, 7K MWCO, Thermo Scientific) twice immediately prior to analysis and sprayed directly into the mass-spectrometer. (A) Mass spectra of TEM-116 incubated with enmetazobactam or tazobactam. Charge states are in red. (B) Magnification of the major charge state (+10) of the respective spectra in (A). Note that in the case of TEM-116 on incubation with both tazobactam and enmetazobactam an additional adduct of +62 Da was observed, both for the unmodified and the dehydrated protein. This species may result from binding of a penam degradation product, i.e. hydrated acetaldehyde. ● Corresponds to **9** or **10** + 62 Da and ◊ corresponds to **1** + 62 Da. (C) Mass spectra of OXA-10 incubated with enmetazobactam, or tazobactam. Charge states are indicated in red. (D) Magnification of the major charge state (+14) of the respective spectra in (C). Proposed structures of the labelled species are given in Figure 2 and Figure S17A. Assignments are shown in Table S5. *Note mass shifts may reflect more than one species, e.g. **9**-**10** give the same mass shift.

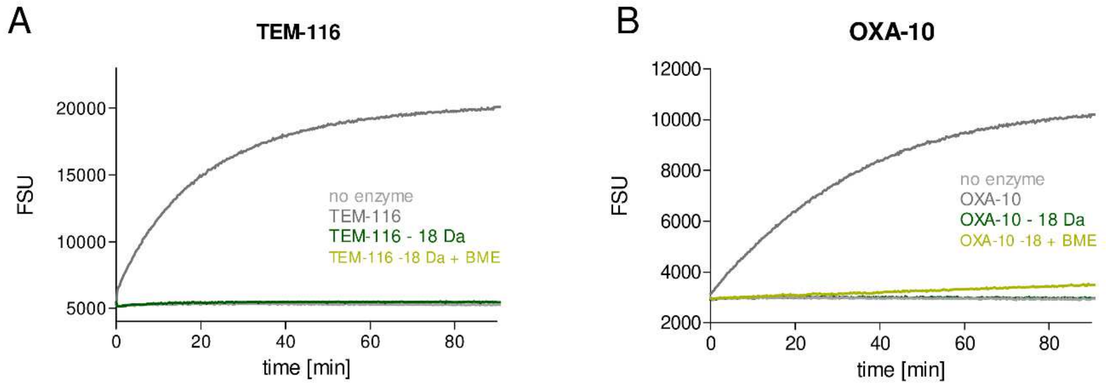

Figure S27: β-Lactamase activity assay for -18 Da modified TEM-116 and OXA-10. Prior to the assay TEM-116 (3 µM) was preincubated either without inhibitor, with enmetazobactam (900 µM), or with a combination of enmetazobactam (300 µM) and β-mercaptoethanol (BME, 3 mM) for 24 h in 50 mM Tris pH 7.5. OXA-10 (3 µM) was preincubated either without inhibitor, with tazobactam (300 µM), or with a combination of tazobactam (300 µM) and BME (3 mM). (A) 100 pM TEM-116 was assayed using 5 µM FC-5(1). (B) 300 pM OXA-10 was assayed using 5 µM FC-5(1). Buffer: 50 mM phosphate buffer, pH 7.0, 7.5, or 8.0 and 0.01% (v/v) Triton X-100. Note, that the residual activity of the OXA-10 -18 Da + BME sample (< 5 % of activity of unmodified OXA-10) may be due to small amounts of unmodified enzyme.

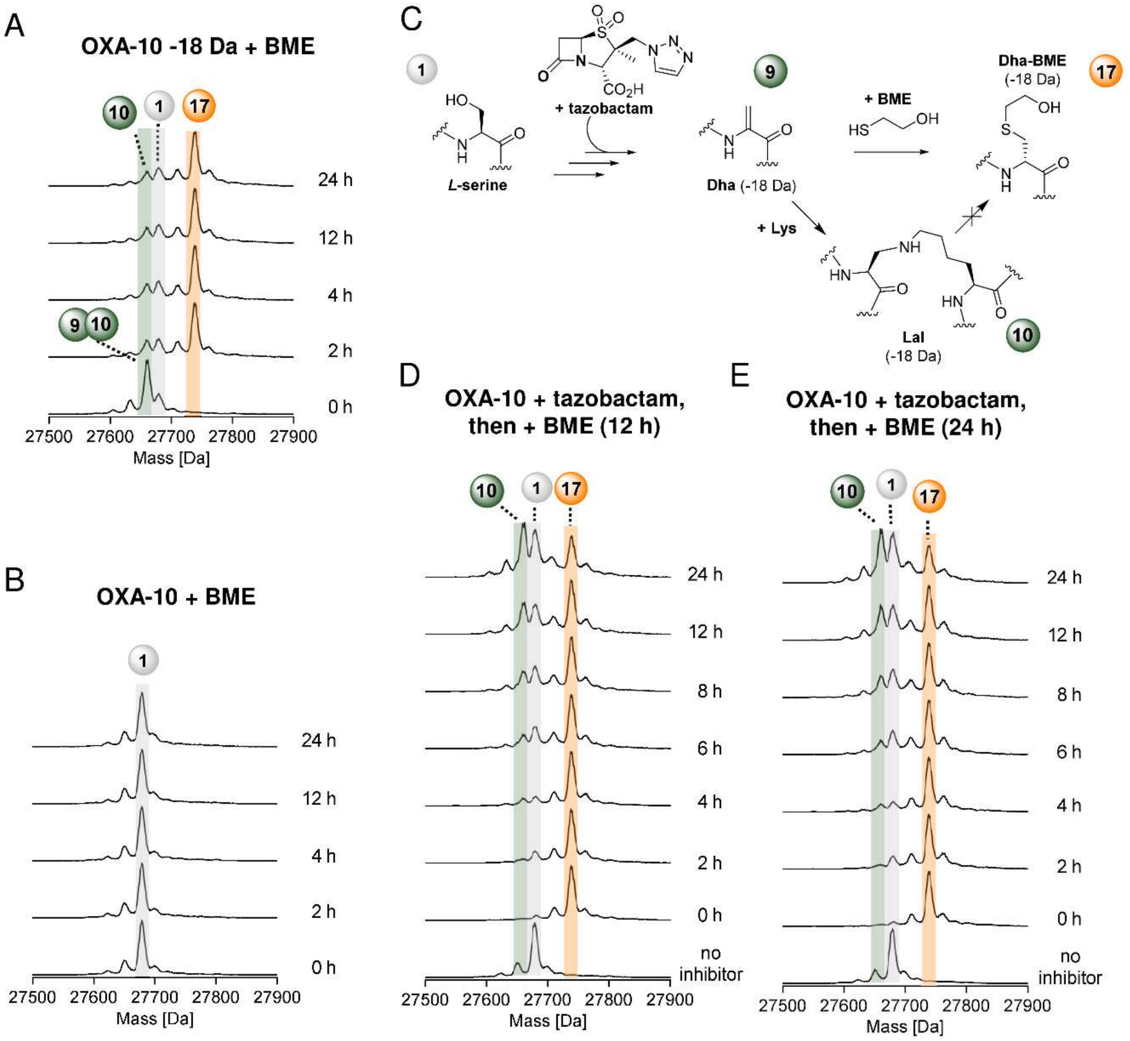

Figure S28: SPE-MS time courses for reaction of the OXA-10 S67Dha modified protein with β-mercaptoethanol (BME). OXA-10 (3 µM) was incubated with tazobactam (300 µM) and BME (3 mM) in 50 mM Tris pH 7.5 at r.t. The order and timing of the additions of tazobactam and BME were varied. (A) OXA-10 incubated with tazobactam leading to apparently complete conversion to the -18 Da modified protein (likely **9** or **10,** Figure 2), before addition of BME at t = 0 h. The results show ~80% addition of BME to the -18 Da modified OXA-10 within 2 h (likely to give **9** or **10**); no further progression of the reaction was observed over an additional 22 h. (B) BME was added to unmodified OXA-10 (in absence of tazobactam) at t = 0 h. No addition of BME to unmodified OXA-10 was observed over the duration of the experiment. (C) Proposed reaction scheme for OXA-10 modification on treatment with tazobactam and BME. (D) OXA-10 was incubated with tazobactam for the indicated time before addition of BME. Samples were then incubated for an additional 12 h with continuous rocking. The results show less addition of BME to the "aged" -18 Da modified protein, consistent with reaction of the S67Dha modified protein **9** to give the S67Dha-K70 Lal protein **10**, which does not react with BME to give an (2-hydroxyethyl)-cysteine residue (Dha-BME) **17**. (E) Samples from (D) measured after 24 h, showing no evidence for further reaction of any of the samples.

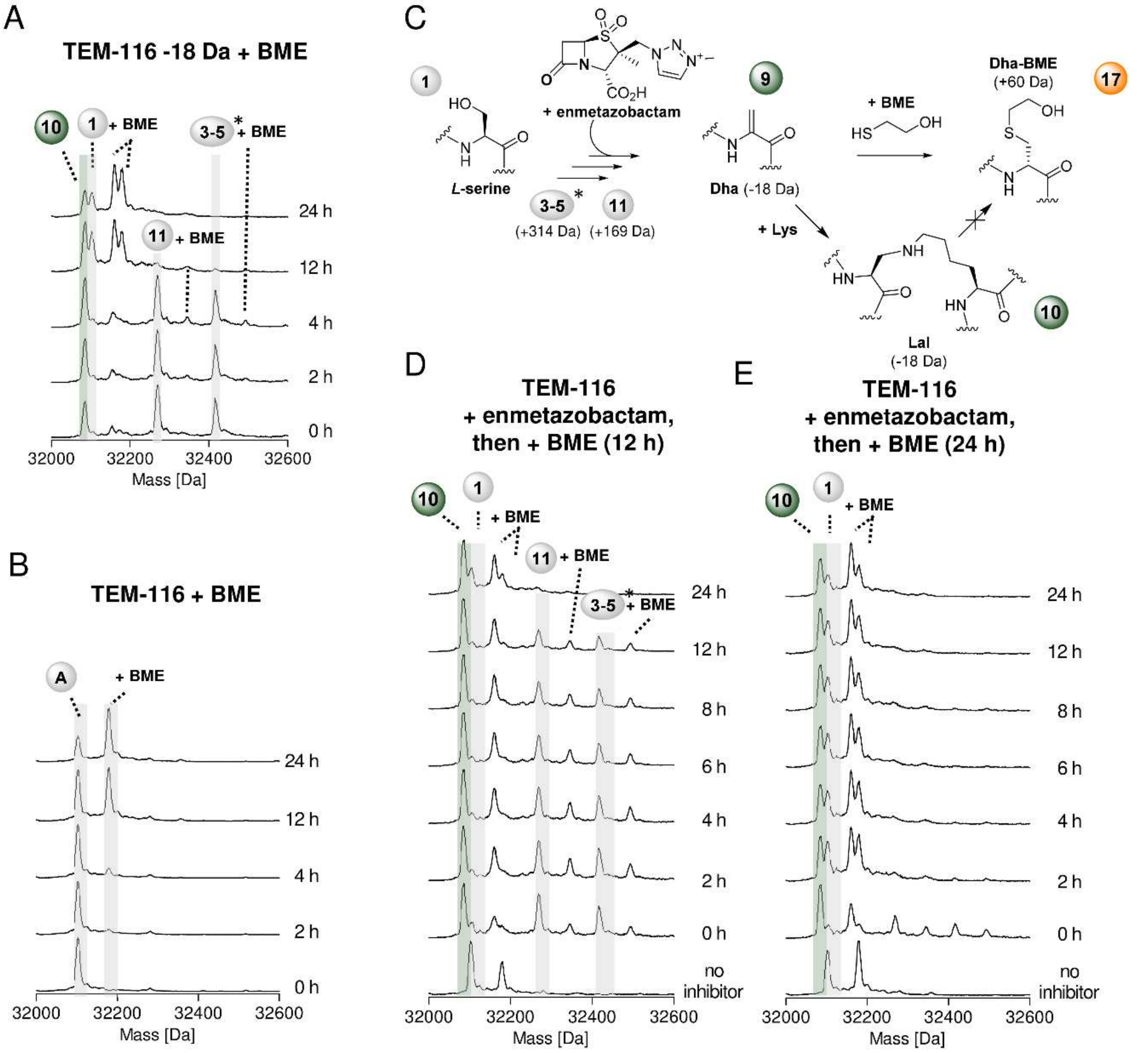

Figure S29: SPE-MS time courses for reaction of TEM-116 S70Dha modified protein with β-mercaptoethanol (BME). Unless stated otherwise, TEM-116 (3 µM) was incubated with enmetazobactam (300 µM) and BME (3 mM) in 50 mM Tris pH 7.5 at r.t. The order and timing of the additions of both enmetazobactam and BME were varied. (A) TEM-116 incubated with enmetazobactam for 8 h leads to partial conversion to the -18 Da modified protein (likely **9** or **10,** Figure 2), BME was then added at t = 0 h. The results indicate addition of BME to the -18 Da modified protein, as well as to unmodified TEM-116 and enmetazobactam modified TEM-116 (**3**-**5** and **11**, or equivalent mass species, Figure 2). (B) BME added to unmodified TEM-116 (without tazobactam) at t = 0 h; addition of BME was observed. (C) Proposed reaction scheme for TEM-116 modification on treatment with enmetazobactam and BME. (D) TEM-116 was incubated with enmetazobactam for the indicated time; BME was then added and the samples were incubated for 12 h. The results show no evidence for addition of a second BME molecule, independent of pre-incubation time with enmetazobactam, suggesting efficient formation of the 70-73 Lal cross-link **10** following the formation of Dha **9**. (E) Samples from (D) measured after 24 h, showing no evidence for addition of a second BME molecule.

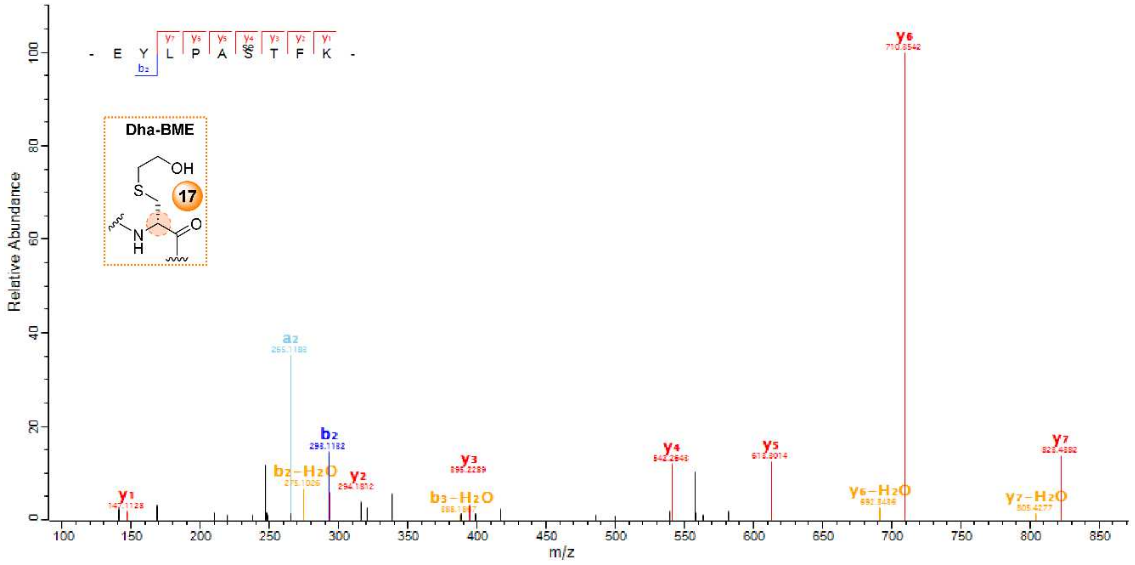

Figure S30: LC-MS/MS analysis of tryptic digest peptide of OXA-10 modified by reaction with tazobactam in the presence of β-mercaptoethanol (BME). OXA-10 (3 µM) was incubated with tazobactam (300 µM) and BME (3 mM) for 24 h in 50 mM Tris pH 7.5 at r. t., then desalted prior to trypsin digestion. MS/MS spectrum of the 62-EYLPASTFK-70 tryptic peptide (MH^{2+} ion), consistent with the Ser64 +60 Da modification **17** (resulting from Dha **9** formation, followed by BME addition to give (2-hydroxyethyl)-*D*-cysteine, Dha-BME **17**).

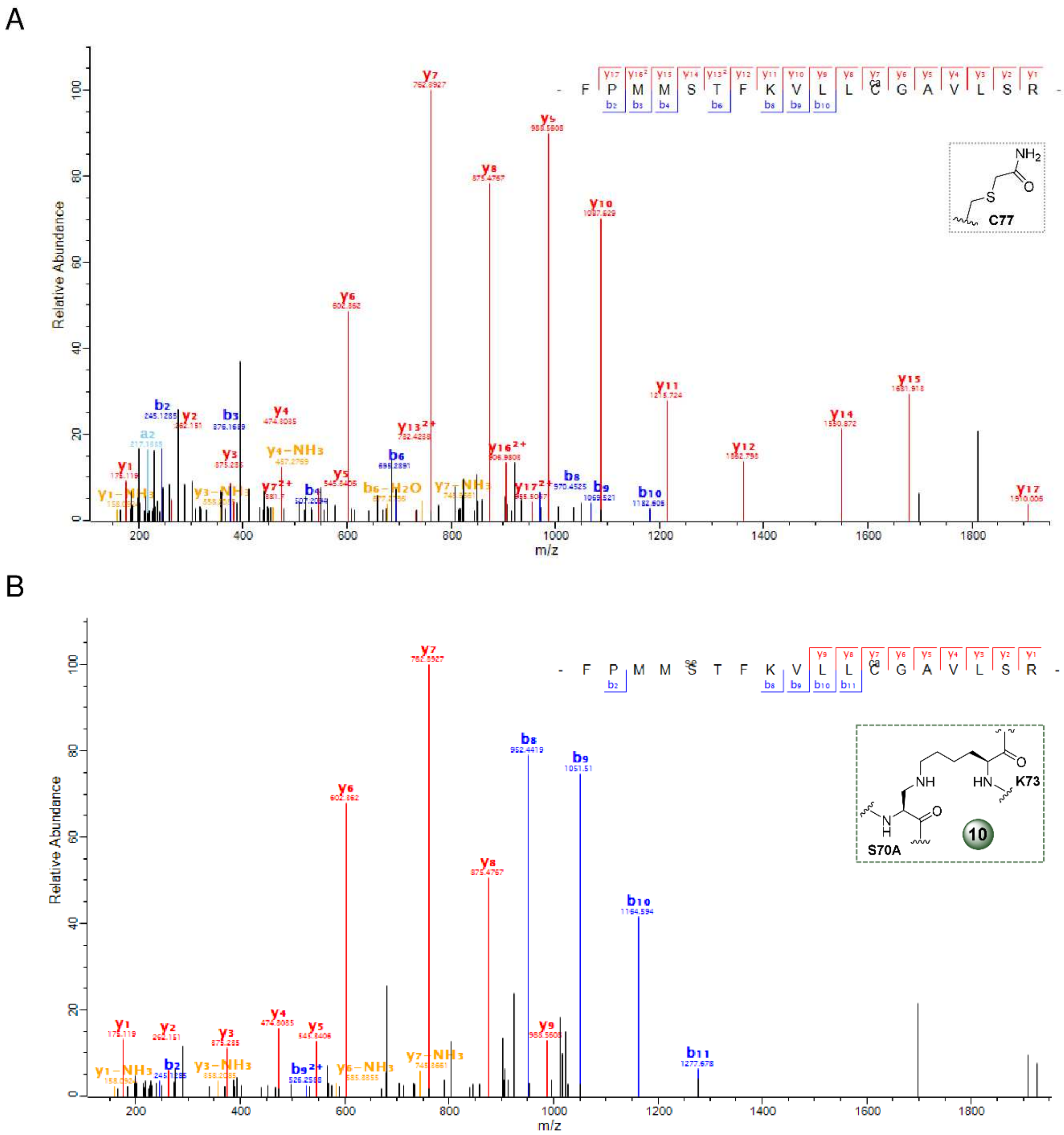

Figure S31: LC-MS/MS analysis of tryptic digest peptides of TEM-116 modified by reaction with enmetazobactam. TEM-116 (3 µM) was incubated with enmetazobactam (300 µM) for 24 h in 50 mM Tris pH 7.5 at r.t., then desalted prior to trypsin digestion. (A) MS/MS analysis of the 66-FPMMSTFKVLLCGAVLSR-83 tryptic peptide (MH^{3+} ion) consistent with an +58 Da acetamide modification at C77. (B) MS/MS analysis of the same peptide sequence with a -18 Da modification (likely **10**).

Table S7: Data collection and refinement statistics for OXA-10 crystals.

Datasets	OXA10-Dha (PDB: 7B3S)	OXA10-Dha-BME (PDB: 7B3U)	OXA10-Lal (PDB: 7B3R)
**Data Collection**			
Beamline (Wavelength, Å)	DLS I24 (0.9686)	DLS I24 (0.9686)	DLS I24 (0.9686)
Detector	Pilatus3 6M	Pilatus3 6M	Pilatus3 6M
Data Processing	Xia2 dials	Xia2 dials	Xia2 dials
Space group	$P\,2_1\,2_1\,2_1$	$P\,2_1\,2_1\,2_1$	$P\,2_1\,2_1\,2_1$
Cell dimensions			
a,b,c (Å)	48.9, 97.2, 125.9	48.9, 97.2, 126.0	48.6, 95.5, 126.2
α, β, γ (°)	90, 90, 90	90, 90, 90	90, 90, 90
No. of molecules/ASU	2	2	2
No. reflections	52193 (2503)*	80334 (3850)*	52627 (2568)*
Resolution (Å)	48.90-1.85 (1.88-1.85)*	76.97-1.60 (1.63-1.60)*	63.09-1.83 (1.86-1.83)*
R_{merge}(I)	0.275 (2.398)*	0.151 (1.185)*	0.182 (1.544)*
I/σI	7.1 (1.2)*	8.9 (1.6)*	8.7 (1.8)*
$CC_{1/2}$	0.99 (0.61)*	1.00 (0.82)*	1.00 (0.85)*
Completeness (%)	100 (97.6)*	99.9 (96.7)*	99.8 (97.7)*
Multiplicity	12.9 (12.9)*	12.7 (11.9)*	12.9 (13.0)*
Wilson B value ($Å^2$)	9.58	14.95	21.27
**Refinement**	PHENIX	PHENIX	PHENIX
R_{work}/R_{free}	0.1649/0.2030	0.1551/0.1767	0.1698/0.1906
No. atoms	4413	4571	4202
- Enzyme	3867	3937	3747
- Ser64Dha	12	-	-
- Ser64Dha-BME	-	18	-
- Ser64Dha-Lys70 crosslink	-	-	60
- Water	507	576	396
Average B-factors	23.86	26.28	32.64
- Enzyme	22.34	24.59	32.12
- Ser64Dha	18.02	-	-
- Ser64Dha-BME	-	18.62	-
- Ser64Dha-Lys70 crosslink	-	-	25.89
- Water	34.75	37.25	38.56
RMS[$] deviations			
- Bond lengths (Å)	0.009	0.006	0.005
- Bond angles (°)	1.004	0.885	0.861

[#] ASU = asymmetric unit.
[$] RMS = root mean square.
*Highest resolution shell in parentheses.

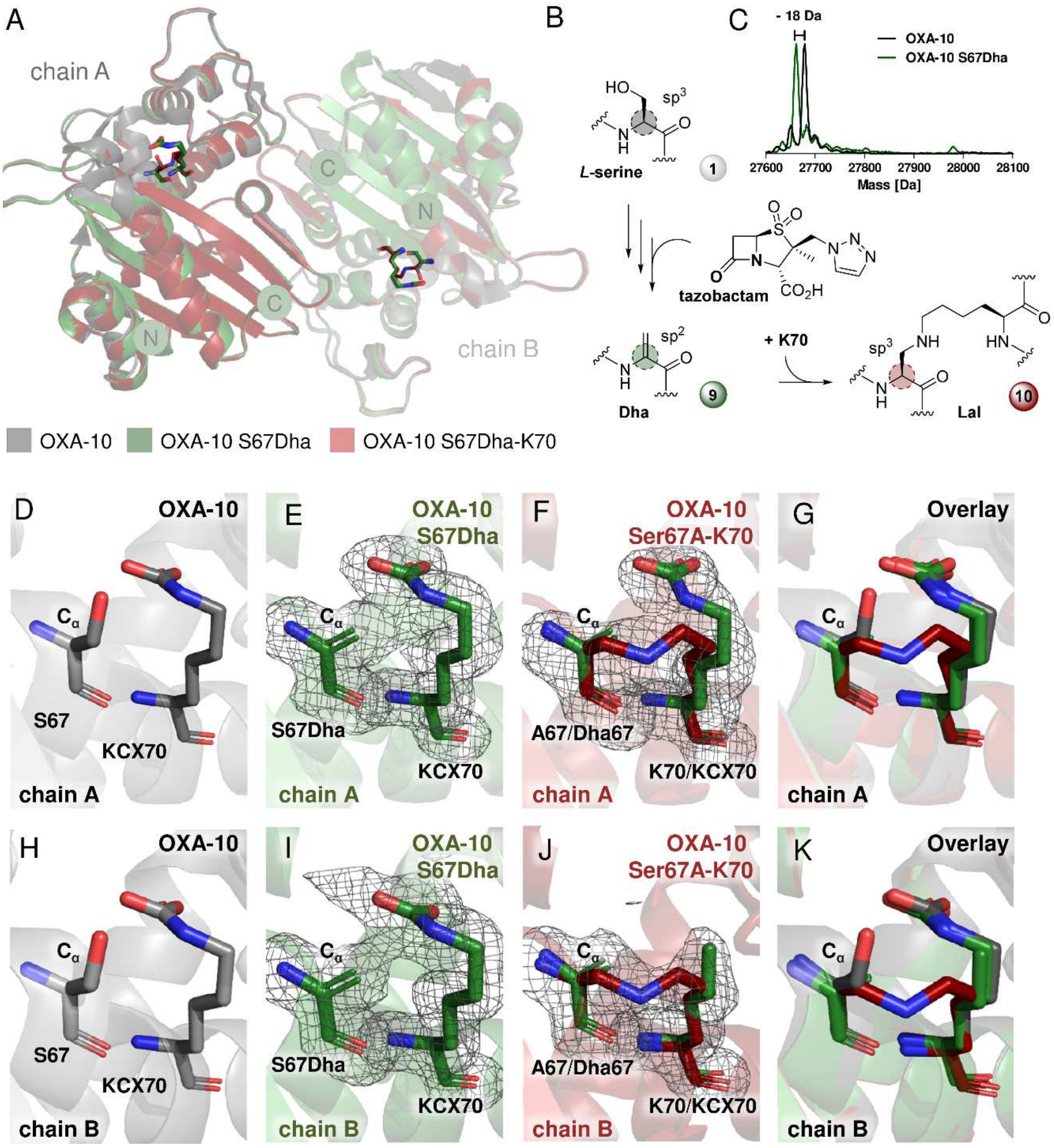

Figure S32: Views from crystal structures of OXA-10 after reaction with tazobactam to produce -18 Da mass shifts. (A) Overview of the fold of unmodified OXA-10 (PDB: 2X02(8), 1.35 Å resolution), OXA-10 S67Dha **9** (PDB: 7B3S, 1.85 Å resolution), and S67Dha OXA-10 in combination with a partial S67A-K70 Lal-crosslinked **10** OXA-10 structure (PDB: 7B3R, 1.83 Å resolution). (B) Reaction of OXA-10 with tazobactam. (C) SPE-ESI MS spectra of OXA-10 before and after modification by reaction with tazobactam. (D-G) Active site views of chain A. (H-K) Active site views of chain B. mF_o-DF_c polder OMIT maps(6) are contoured at 3.0 σ and carved around active site residues.

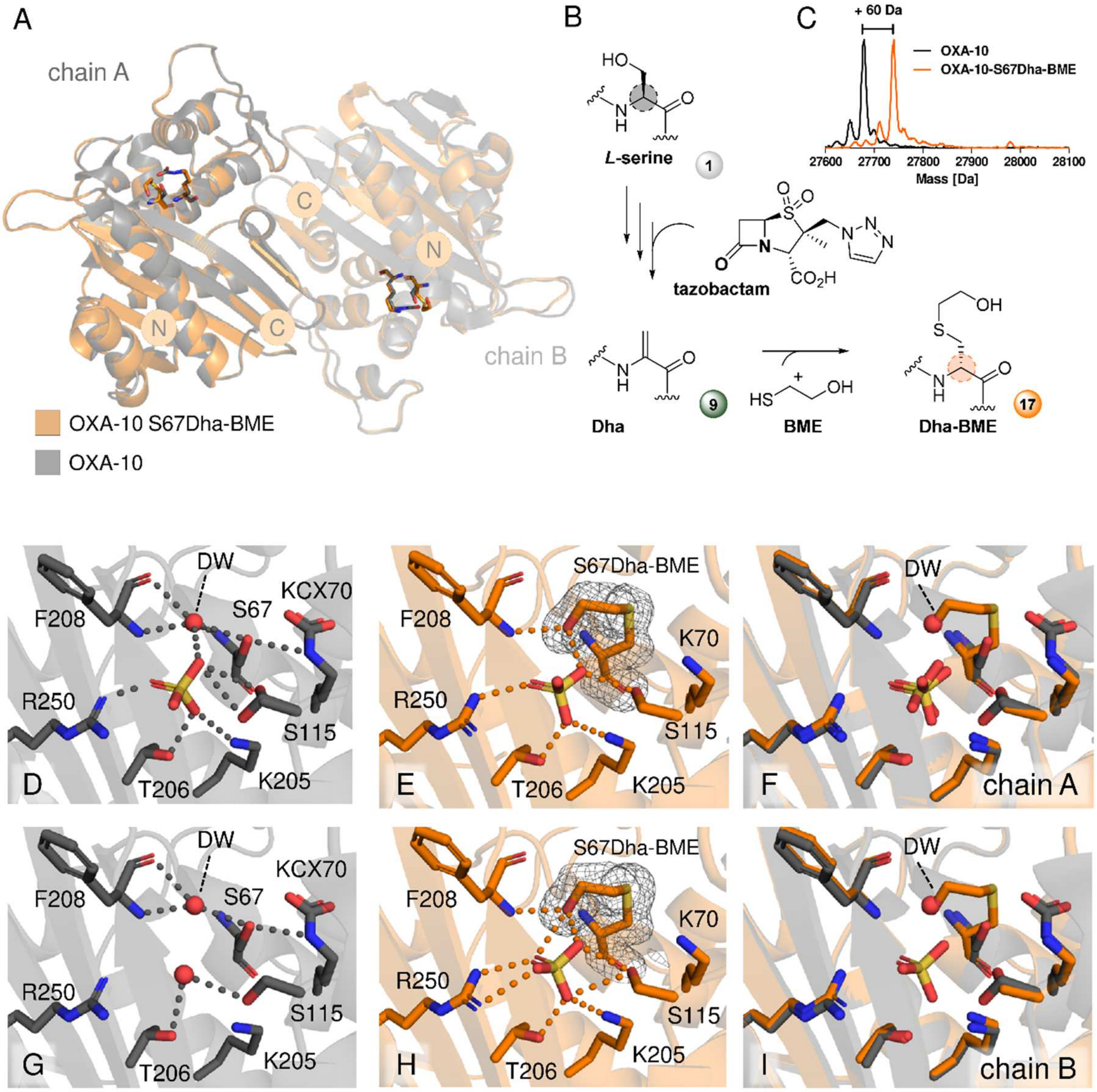

Figure S33: Views from a crystal structure of OXA-10 S67Dha reacted with β-mercaptoethanol (BME) to give (2-hydroxyethyl)-*D*-cysteine (Dha–BME). (A) Overview of OXA-10 fold of unmodified OXA-10 (PDB: 2X02(8), 1.35 Å resolution) and OXA-10 S67Dha-BME **17** (PDB: 7B3U, 1.60 Å resolution). (B) Reaction scheme for OXA-10 reaction with tazobactam, then BME. (C) SPE-ESI MS spectra of OXA-10 before and after reaction with tazobactam, then BME. (D-F) Active site interactions of chain A of unmodified OXA-10 (D), S67Dha-BME (E) mF_o-DF_c polder OMIT maps(6) contoured at 3.0 σ, carved around S67Dha-BME. (F) Superimposition of unmodified and S67Dha-BME **17** active site views. (G-I) Active site interactions of chain B of unmodified OXA-10 (G) and S67Dha-BME (H). (I) Superimposition of the unmodified and S67Dha-BME **17** active site views. Note that even though the generation of small amounts of the *L*-isomer cannot be excluded, the maps indicate formation of a (2-hydroxyethyl)-*D*-cysteine residue. In both chains the hydroxyethyl oxygen is oriented so that the OH is positioned in the same location as the deacylating water (DW) in the active site of unmodified OXA-10.

References

1. S.S. Van Berkel*, et al.*, Assay platform for clinically relevant metallo-β-lactamases. *J. Med. Chem.* 56, 6945-6953 (2013).
2. K.M. Papp-Wallace*, et al.*, Beyond Piperacillin-Tazobactam: Cefepime and AAI101 as a Potent β-Lactam-β-Lactamase Inhibitor Combination. *Antimicrob. Agents Chemother.* 63, e00105-00119 (2019).
3. F. Danel, J.-M. Frère, D.M. Livermore, Evidence of dimerisation among class D β-lactamases: kinetics of OXA-14 β-lactamase. *Biochim. Biophys. Acta* 1546, 132-142 (2001).
4. C. Chang, Maltseva, N., Endres, M., Joachimiak, A., Center for Structural Genomics of Infectious Diseases (Csgid), Class C β-lactamase from *Escherichia coli* in complex with Tazobactam. 10.2210/pdb6wa7/pdb (2020).
5. P.A. Lang*, et al.*, Bicyclic Boronates as Potent Inhibitors of AmpC, the Class C β-Lactamase from *Escherichia coli*. *Biomolecules* 10, 899 (2020).
6. D. Liebschner*, et al.*, Polder maps: improving OMIT maps by excluding bulk solvent. *Acta Crystallogr. Sect. D. Biol. Crystallogr.* 73, 148-157 (2017).
7. G.J. Poelarends, W.H. Johnson, Jr., A.G. Murzin, C.P. Whitman, Mechanistic characterization of a bacterial malonate semialdehyde decarboxylase: identification of a new activity on the tautomerase superfamily. *J. Biol. Chem.* 278, 48674-48683 (2003).
8. L. Vercheval, Kerff, F., Sauvage, E., Herman, R., Galleni, M., Charlier, P., Crystal structure of the class D β-lactamase OXA-10 at 1.35 Å resolution. 10.2210/pdb2X02/pdb (2010).
